# IFI27 and BAX are Essential for GSDME‐Mediated Myeloma Cell Pyroptosis

**DOI:** 10.1002/advs.77164

**Published:** 2026-08-30

**Authors:** Yaoli Cui, Yuening Sun, Ziyang Liu, Xuntao Lai, Yunshu Zhao, Yan'er Wang, Yuanming He, Fang Xu, Du Feng, Longlong Liu, Zhenqian Huang, Lijie Xing, Xinliang Mao

**Affiliations:** ^1^ Department of Hematology The Key Laboratory of Advanced Interdisciplinary Studies The First Affiliated Hospital of Guangzhou Medical University Guangzhou People's Republic of China; ^2^ Guangdong Provincial Key Laboratory of Protein Modification and Degradation School of Basic Medical Sciences Guangzhou Medical University Guangzhou People's Republic of China; ^3^ Department of Lymphoma Shandong Cancer Hospital and Institute Shandong First Medical University and Shandong Academy of Medical Sciences Jinan Shandong People's Republic of China

**Keywords:** gasdermin E, interferon alpha‐inducible protein 27, mitochondria, multiple myeloma, pyroptosis

## Abstract

Induction of pyroptosis is a novel strategy for multiple myeloma (MM) treatment, but the underlying mechanism remains elusive. In the analysis of the transcriptomic profile in pyroptotic MM cells, we find the interferon‐alpha inducible protein IFI27 is strikingly upregulated. IFI27 is downregulated in MM cells in association with poor prognosis, but its overexpression displays great potency to trigger pyroptosis in both MM cell lines and newly diagnosed MM cells in a GSDME‐dependent manner. Moreover, ectopic IFI27 impairs mitochondrial structure and function. Interestingly, when mitochondria are depleted, IFI27 almost fails to induce MM cell pyroptosis. Mechanistic studies show that IFI27 recruits N‐GSDME to mitochondria via BAX, therefore triggering pyroptosis. When IFI27 is knocked down, MM cells hardly undergo pyroptosis even when triggered by N‐GSDME, BAX, or chemotherapeutic agents. Although GSDMD induces cell pyroptosis independent of BAX, BAX is required for IFI27‐ and N‐GSDME‐induced cell pyroptosis. Lastly, ectopic IFI27 promotes GSDME activation and triggers MM cell pyroptosis in vivo and strikingly prolongs the survival of mice with MM. In summary, the present study finds that IFI27 and BAX are essential for GSDME‐mediated cell pyroptosis, and induction of IFI27 may represent a promising strategy for the treatment of MM expressing GSDME.

## Graphical abstract

The proposed model of GSDME‐mediated MM cell pyroptosis modulated by IFI27 and BAX. IFI27 is downregulated in MM cells. Upon pyroptotic stimulation, such as DOX or ETO treatment, IFI27 is induced, therefore liberating BAX from BCL‐2 ❶. BAX is then recruited to mitochondria and forms homo‐oligomeric channels in OMM, therefore leading to cytochrome C leaks to the cytosol ❷ and activating Caspase‐3 ❸, which further cleaves and activates GSDME ❹❺. The resultant N‐GSDME is further identified by BAX ❻ and is recruited to OMM by IFI27 ❼. N‐GSDME forms pores in OMM and also leads to cytochrome C release ❽, therefore further activating Caspase‐9/Caspase‐3/GSDME ❾. More N‐GSDME fragments then form pores in the plasma membrane, leading to cell pyroptosis ❿. The upregulated IFI27, liberated BAX, and activated GSDME collaborate in mitochondrial structural damage and dysfunction that further exaggerates MM cell pyroptosis.

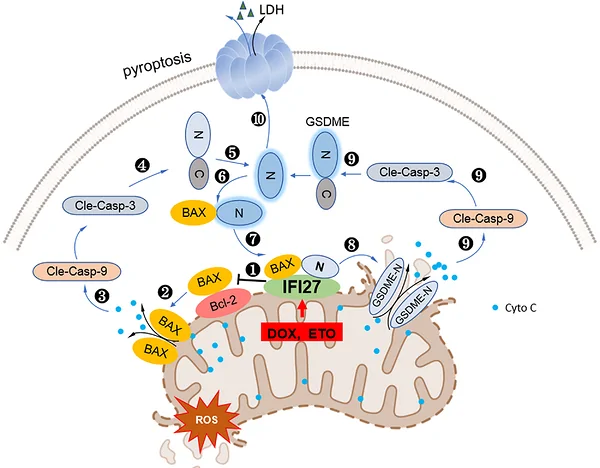

## 1 Introduction

The malignant plasma cell disorder multiple myeloma (MM) is the second most common hematological cancer, mainly seen in seniors but also reported in young populations under 30 years old [1]. Current treatments include targeted therapies, chemotherapies, bone marrow transplantation, as well as monoclonal antibodies and chimeric antigen receptor T (CAR‐T) cell therapy, and these advanced comprehensive modalities have significantly improved the overall survival (OS) and progression‐free survival (PFS), but the overall five‐year survival rate is not satisfactory [2]. Therefore, it is urgent to develop novel forms of therapeutic strategy for MM patients.

Pyroptosis is a novel form of programmed cell death that utilizes a group of proteins called gasdermins as executors, specifically GSDM‐A, ‐B, ‐C, ‐D, and ‐E. Triggered by pyroptotic signaling, gasdermins are cleaved and activated by specific caspases or granzymes, and the resultant N‐terminal fragments form pores in the plasma membrane, therefore leading to increased permeabilization and cell death [3]. Induction of pyroptosis has thus been proposed as a promising novel strategy for the treatment of cancers, including hematological malignancies [3]. Recent studies demonstrate that MM cells undergo pyroptosis induced by various agents, including bortezomib [4], clioquinol [5], and acevaltrate [6]. Moreover, although mitochondria are important for pyroptosis, GSDMD is reported to induce cell pyroptosis via cardiolipin rather than BAX and BAK [7], two key factors in maintaining mitochondrial structure and function, in inflammatory cells, including macrophages. The underlying mechanisms for GSDME translocalization to mitochondria are yet to be elucidated, and important therapeutic targets remain elusive in MM cell pyroptosis.

Interferons (IFNs), a group of secreted cytokines involved in the upregulation of immune responses, have been widely used to treat viral infections and cancers, including MM. A meta‐analysis of 17 clinical trials shows that IFN significantly improves clinical outcomes of MM patients, including induction response, relapse‐free, and overall survival, but the detailed mechanisms remain unknown [8]. IFNs are able to stimulate the expression of a large number of genes called interferon‐stimulated genes (ISGs), of which IFIT1 and IFIT3 are able to enhance myeloma cell pyroptosis induced by clioquinol [5]. But whether ISGs are able to directly induce MM cell pyroptosis is not known.

In the present study, we found that a batch of ISGs is upregulated in pyroptotic MM cells treated with doxorubicin (DOX) and etoposide (ETO), two well‐known pyroptosis inducers [9] and drug options for MM treatment [10], and IFI27 is the most striking one. IFI27 is a type I interferon α‐inducible and mitochondrial resident protein [11]. We find that IFI27 is downregulated in MM cells, but its overexpression triggers pyroptosis in cell lines and primary cells from newly diagnosed MM patients. Mechanistically, IFI27 in collaboration with BAX recruits N‐GSDME to mitochondria, therefore inducing GSDME‐mediated and mitochondria‐dependent pyroptosis. Furthermore, the high level of IFI27 expression predicts good outcomes for MM patients, and induction of IFI27 prolongs the survival rate of mice with MM.

## 2 Materials and Methods

### 2.1 Cell Culture

Cells were cultured in Iscove's modified Dulbecco's medium (IMDM) supplemented with 10% fetal bovine serum (Vazyme Ltd., Nanjing, China). Bone marrow specimens were voluntarily donated by MM patients with written consent in accordance with the Helsinki Declaration principles, and this study was approved by the Medical Ethics Board of the First Affiliated Hospital of Guangzhou Medical University (Approval No. #ES‐2024‐201‐01).

### 2.2 Plasmids

The human pWPI‐GSDME and pWPI‐GSDME‐D270A plasmids were kindly provided by Dr. Feng Shao (National Institute of Biological Sciences, Beijing, China). A Myc‐Parkin plasmid was maintained by the lab. The mCherry‐IFI27 (wild type) was provided by IGE Biotechnology LTD (Guangzhou, China). The truncates were constructed with Mut Express II Fast Mutagenesis Kit V2 (Cat. #C214, Vazyme). The pLV3‐Flag‐IFI27, the Tet‐on/IFI27, and the Tet‐on/IFI27‐GFP lentivirus plasmids were from Miaoling Biotechnology LTD (Wuhan, China). The N‐GSDME fragment was generated from the full‐length one and cloned into a pCDH vector carrying a Flag tag or cloned into a pcDNA3.1 vector carrying a Myc tag. The full‐length IFI27 and BAX were cloned from OCI‐My5 cells and inserted into a pCDH‐eGFP lentiviral vector. The detailed primers for each plasmid were described in the Supplemental File.

### 2.3 CRISPR‐Cas9 Knockout (KO) and the Lentiviral System

The KO cell lines were generated by using the CRISPR/Cas9 technology [4]. Details were described in the supplemental protocol. The detailed single guide (sg) RNAs for IFI27, BAX and GSDME were described as follows: The oligonucleotide sequences of sgGSDME were: sgRNA‐GSDME#1, 5’‐GTATAACTCAATGACACCGT‐3’, sgRNA‐GSDME#2, 5’‐GAGAAGTGTGGTGGCATCGT‐3’, sgRNA‐IFI27#1, 5’‐ACAACTGTAGCAATCCTGGC‐3’, sgRNA‐IFI27#2, 5’‐ATTGCTACAGTTGTGATTGG‐3’; sgRNA‐BAX#1, 5’‐CGGGATCCTTCCTACCGGCC‐3’, sgRNA‐BAX#2, 5’‐GGGGCAGAAACTCCCGGATC‐3’.

### 2.4 Lactate Dehydrogenase (LDH) Release Assay

LDH in culture media was measured by using the LDH Cytotoxicity Assay Kit (Beyotime) following the manufacturer's instructions as described previously [4].

### 2.5 RNA Sequencing

Total RNAs from cells of interest were extracted with an RNA isolation kit as shown previously [5]. The prepared RNA samples (n = 3) were subjected to transcriptomic sequencing by IGE Biotechnology LTD (Guangzhou, China).

### 2.6 Mitochondria Isolation, Intracellular ROS, and Mitochondrial Membrane Potential (MMP) Assays

Mitochondria were isolated using the Cell Mitochondria Isolation Kit (Cat. #C3601, Beyotime) as instructed by the manufacturer. The ROS levels were measured using the DCFH‐DA reagent from the ROS Assay Kit (Cat. #S0033S, Beyotime); analysis of MMP was carried out by using the Mitochondrial Membrane Potential Assay Kit with TMRE (Cat. #C2001S, Beyotime). The detailed protocols were described previously [4, 6].

### 2.7 Immunofluorescent Assay

Before staining with specific antibodies, cells were treated and fixed as described previously [12]. Cell nuclei and mitochondria were stained with Hoechst33342 and MitoTracker, respectively, before being analyzed on a Zeiss LSM 900 confocal microscope.

### 2.8 Depletion of Mitochondria

To deplete mitochondria via enforced mitophagy, cells of interest were stably expressing Myc‐Parkin by lentiviral infection followed by treatment with 20 µm CCCP (carbonyl cyanide m‐chlorophenyl hydrazone) when cells reached 90%–95% confluency [13]. The efficiency of mitochondrial depletion was determined by detecting mitochondrial proteins and MitoTracker staining.

### 2.9 Transmission Electron Microscopy (TEM)

Cells were processed before TEM analysis as described previously [14]. Subcellular structure analysis was performed on a Hitachi HT7700 TEM (Tokyo, Japan).

### 2.10 Seahorse Cell Mitochondrial Stress Test Assay

Mitochondrial respiration capacity of MM cell lines infected with IFI27 (Tet‐on) lentivirus was detected by Seahorse Cell Mito Stress Test Assay [15]. The Seahorse XFe96 Sensor Cartridge was hydrated overnight at 37°C in a non‐CO_2_ incubator using XF calibrant (Cat. #100840‐000, Sigma). The detailed protocol followed the manufacturer's instructions.

### 2.11 In Vivo Xenograft Models

Two MM xenograft models were established. The subcutaneous model was derived from OCI‐My5 and OPM2 cells stably expressing Tet‐on/IFI27 at a density of 2 × 10^6^ cells per subcutaneous injection. The mice bearing tumors were randomly assigned into two groups; each was administered with 3% sucrose solution only or containing doxycycline (DXC) (3.2 mg/mL) [16]. The drinking water was changed every two days. Tumor sizes and body weights were monitored daily. The second model was generated by tail‐vein injection of OCI‐My5 and OPM2 cells expressing green fluorescent protein Tet‐on/IFI27‐GFP. Mice were received DXC or sucrose solution only up to 70 days to evaluate mice survival. Severe combined immunodeficient Balb/c mice (female, 5–6 weeks old) were provided by Beijing Vital River Laboratory Animal Technology Co., Ltd. (Beijing, China). These xenograft studies were approved by the Review Board for Animal Welfare and Ethics of Guangzhou Medical University (Approval No. GY2024‐722).

### 2.12 Statistics

Statistical difference between the control and the experimental groups was analyzed by Student's *t*‐test with GraphPad Prism9 (GraphPad Software). *p* < 0.05 was considered statistically significant. No statistical method was used to predetermine the sample size. No data were excluded from the analyses. All studies were independently repeated at least three times.

## 3 Results

### 3.1 IFI27 is Upregulated in MM Cells by Pyroptosis‐Inducing Agents Against Multiple Myeloma

**FIGURE 1:**
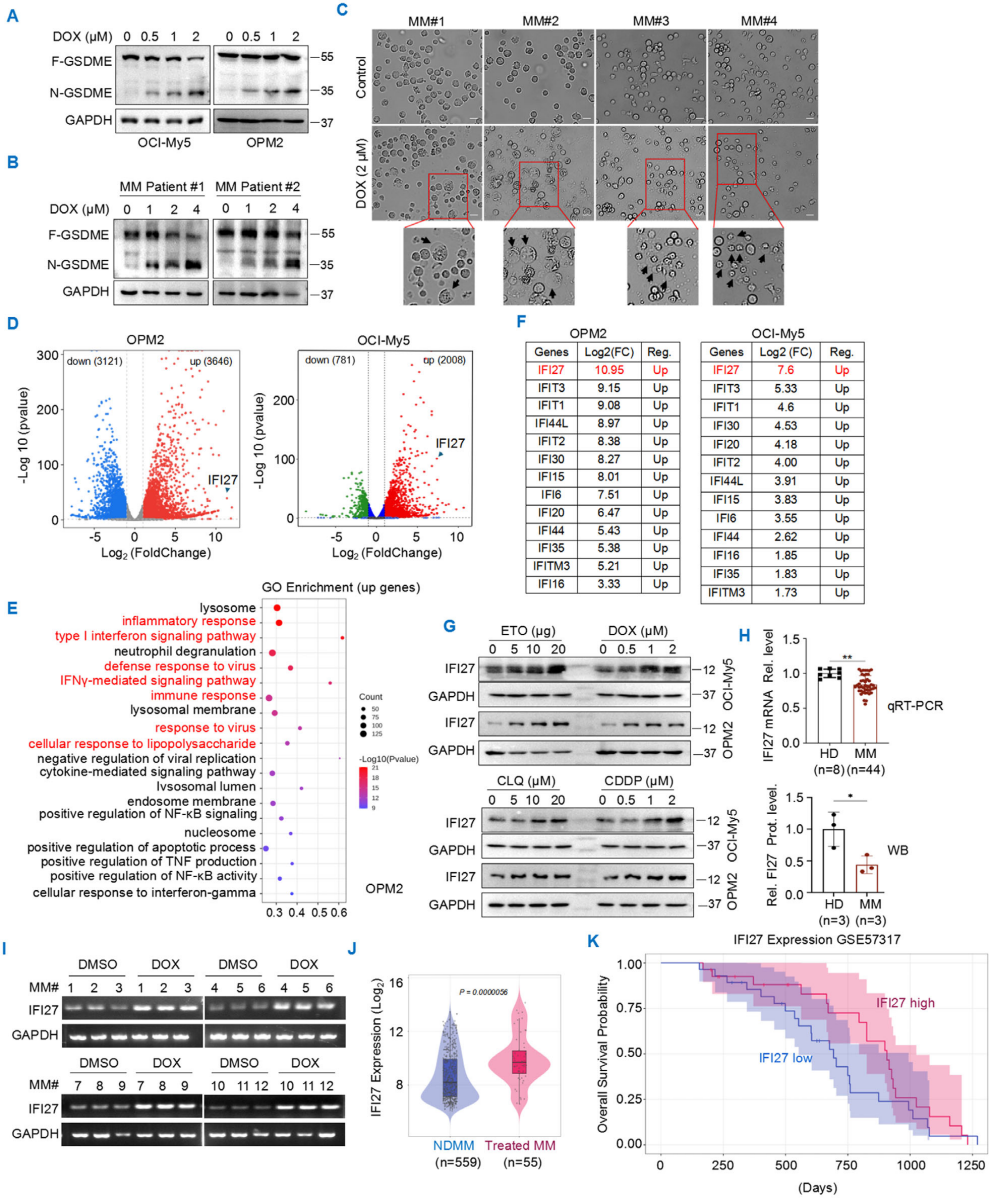
IFI27 is upregulated by pyroptosis‐inducing agents against multiple myeloma. (A) MM cell lines were treated with DOX at indicated concentrations for 24 hr, followed by cell lysate preparation and IB assays. (B, C) MM patient cells were treated with DOX for 24 h. IB assays (B) and phase‐contrast imaging assays (C) were performed. Arrowheads indicated balloon‐like pyroptotic cells; scale bar, 20 µm. (D–F) OCI‐My5 and OPM2 cells were treated with DOX (2 µm) for 24 h, cells were then prepared for RNA‐seq (n = 3), followed by the volcano plot analysis (D), the GO enrichment analysis (E), and the expression of interferon‐induced genes (F). G, OCI‐My5 and OPM2 cells were treated with different anti‐cancer drugs as indicated for 24 h, followed by IB assays. (H) Isolated bone marrow cells from MM patients or healthy donors (HD) were collected, followed by qRT‐PCR and WB assay analyses as indicated. (I) Isolated bone marrow cells from MM patients were treated with DOX for 24 h, followed by RT‐PCR analyses as indicated. (J) Gene expression differences between Naive (untreated, n = 559) and Treated (n = 55) patients were compared based on the GSE57317 and GSE24080 datasets. (K), IFI27 expression and clinical outcomes were evaluated based on the GSE57317 data. IFI27 predicted a positive prognosis for patients with MM.

Chemotherapeutic agents such as doxorubicin (DOX) have been demonstrated to induce pyroptosis in cancer cells [9]. We found that DOX also induced pyroptosis in both MM cell lines and primary MM cells (Figure 1A–C), but the underlying mechanisms were not clear. To explore the specific mechanism, these cell lines were treated with DOX for 24 h before being subjected to transcriptomic sequencing. The Gene Ontology (GO) Enrichment Analysis revealed that several pathways, including anti‐inflammatory, anti‐viral, and type I interferon signaling pathways, were significantly upregulated and were listed at the top (Figure 1D,E). Among these genes, IFI27 ranked at the top (Figure 1F). Interestingly, our recent study found that IFIT1 and IFIT3, the second and third highest upregulated, can enhance myeloma cell pyroptosis induced by acevaltrate [6] and clioquinol [5], but the role of IFI27 remained elusive in pyroptosis. Therefore, it was chosen for further investigation. The upregulation of IFI27 was confirmed in MM cells treated with other agents, including ETO, cisplatin (CDDP), and clioquinol (CLQ) (Figure 1G). Moreover, IFI27 was markedly downregulated in primary MM cells compared with the healthy controls (Figure 1H), but it was strikingly induced by DOX (Figure 1I). IFI27 was also strikingly upregulated in MM cells derived from treated patients assayed from the public datasets GSE57317 and GSE24080 datasets (Figure 1J). The higher the expression of IFI27, the better the prognosis of MM patients based on the GSE57317 dataset (Figure 1K). Therefore, IFI27 is downregulated in MM, but it is strikingly upregulated after treatment and predicts favorable outcomes.

### 3.2 IFI27 Induces MM Cell Pyroptosis in a GSDME‐Dependent Manner

**FIGURE 2:**
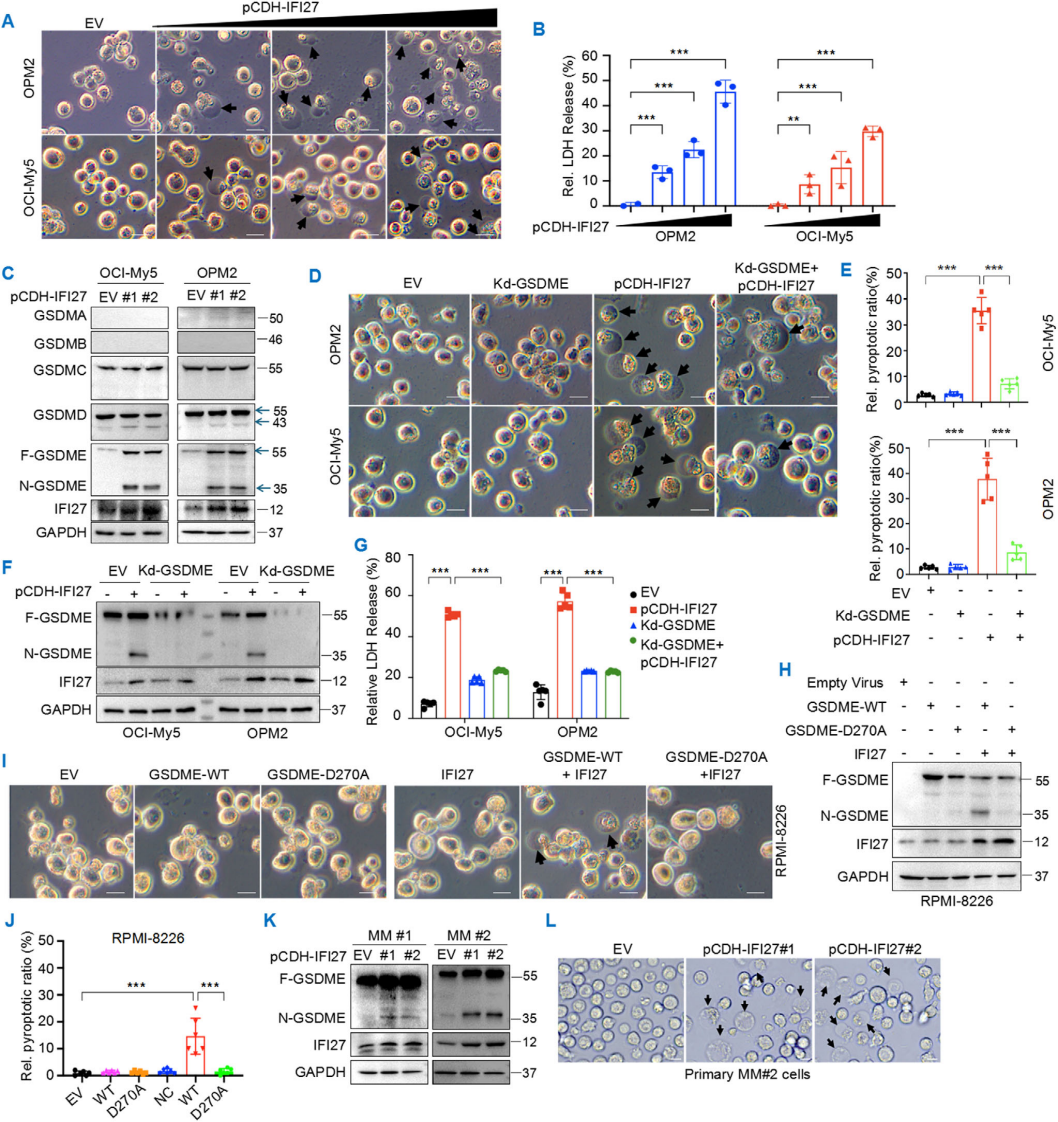
IFI27 induces myeloma cell pyroptosis in a GSDME‐dependent manner. (A, B) OCI‐My5 and OPM2 cells were infected with increased IFI27 lentivirus as indicated, followed by pyroptosis morphology determination (A), and culture media were subjected to LDH measurement (B) (n = 3), after 72 h of overexpression of IFI27 with a concentration gradient. Scale bar, 20 µm. (C) Myeloma cell lines were infected with IFI27 lentivirus for 72 h before cell lysates were prepared for IB assays against different GSDMs. (D–G) GSDME was stably knocked down from OCI‐My5 and OPM2 cells by its specific sgRNA, followed by infection with IFI27 lentivirus. Seventy‐two h later, cells were performed to phase‐contrast microscopy analysis (D) and statistical analysis on pyroptotic cells (E); cell lysates were prepared for IB assays (F) and culture media were subjected to LDH release measurement (G) (n = 5). Scale bar, 20 µm. (H–J) RPMI‐8226 cells were infected with WT or D270A GSDME lentivirus alone or with IFI27 lentivirus for 72 h for IB assays (H), pyroptotic analysis on phase‐contrast microscopy (I), and statistical analysis of pyroptosis rate of cells (J) (n = 6). Scale bar, 20 µm. (K, L) Primary myeloma cells were infected with IFI27 lentivirus for 72 h followed by IB assays using cell lysates (K) and phase‐contrast microscopy assays (L). Scale bar, 10 µm. ^**^, *p* < 0.01; ^***^, *p* < 0.001. EV, empty virus.

Given that IFI27 was strikingly induced by pyroptosis inducers, we wondered whether IFI27 could trigger pyroptosis. To this end, MM cells were infected with lentiviral IFI27 followed by pyroptosis evaluation. The results showed that IFI27 led to typical pyroptotic morphology and LDH release (a hallmark of pyroptosis) [7, 9] in a concentration‐dependent manner (Figure 2A,B). Given that there are five known pyroptotic executors, to find out which one mediated IFI27‐induced MM cell pyroptosis, we examined all five known pyroptosis executors after IFI27 infection. It showed that GSDM‐A and ‐B were not expressed in these MM cells, and IFI27 promoted the cleavage of GSDM‐D and GSDM‐E but not GSDMC (Figure 2C). However, the product of GSDMD cleavage was 43 kDa, which was demonstrated as an apoptotic hallmark and an indicator of GSDMD inactivation [17]. Therefore, GSDME might be the only executor in IFI27‐triggered MM cell pyroptosis. To validate this hypothesis, GSDME was knocked down from OPM2 and OCI‐My5 cells, followed by infection with lentiviral IFI27. The results showed that when GSDME was knocked down, IFI27 failed to induce MM cell pyroptosis (Figure 2D–G). Consistent with these findings, when WT‐GSDME but not the D270A mutant was introduced into RPMI‐8226, a GSDME‐lacking but GSDMD‐expressing MM cell line [4, 5], IFI27 triggered pyroptosis (Figure 2H–J). IFI27 also induced marked pyroptosis in primary cells from MM patients (Figure 2K,L). All these studies thus demonstrate that IFI27 triggers MM cell pyroptosis in a GSDME‐dependent manner.

### 3.3 IFI27 Localization to the Outer Mitochondrial Membrane Is Required for Its Activity to Induce Cell Pyroptosis

**FIGURE 3:**
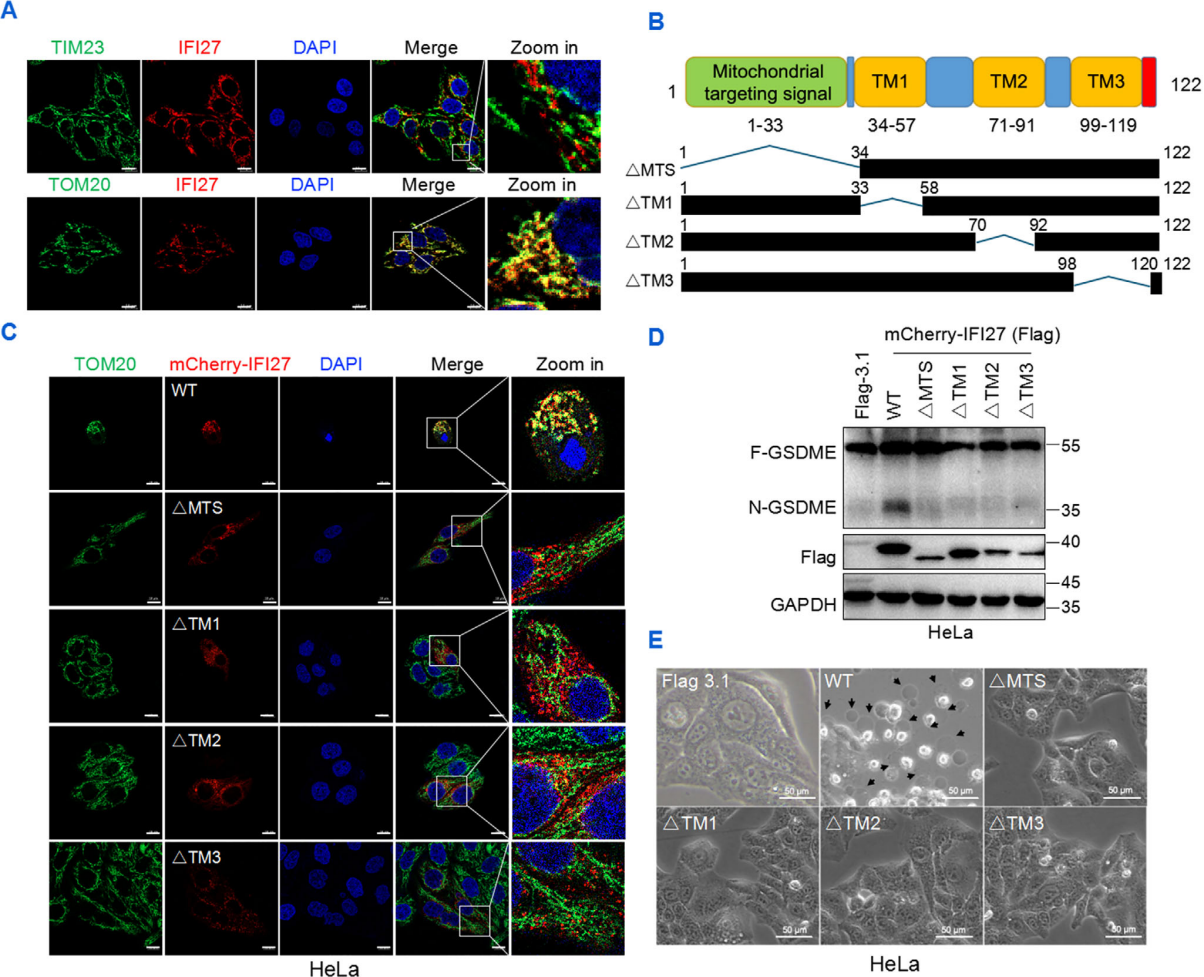
IFI27 localization to the outer mitochondrial membrane is required for its activity to induce cell pyroptosis. (A) Representative confocal microscopy fluorescence images of IFI27 (red) and TOM20/TIM23 (green) in HeLa cells showing that IFI27 was localized in the mitochondrial outer membrane but not inner membrane. Scale bar, 10 µm. (B) IFI27 truncates were constructed based on the schematic representation of the structure of IFI27. (C) HeLa cells were transfected with mCherry‐Flag‐IFI27 wild type or specific truncated plasmids for 44 h, followed by MG132 (20 µm) treatment for another 4 h, followed by fluorescence image analysis (C), IB assays (D), and phase‐contrast microscopy (E). A, C, Scale bars, 10 µm. (E) Scale bar, 50 µm.

Our recent studies showed that other IFIGs, including IFIT1 and IFIT3, are associated with MM cell pyroptosis but can't induce MM cell pyroptosis [5, 6], in contrast, IFI27 induces MM cell pyroptosis, which indicates the specific structure of IFI27 might make the difference. Given that IFI27 is a mitochondrial transmembrane resident protein [11], we wondered whether this specific subcellular localization is critical for IFI27 to induce cell pyroptosis. To validate this hypothesis, we first analyzed subcellular mitochondrial localization and found that IFI27 was co‐expressed with the outer mitochondrial membrane (OMM) protein TOM20 but not the inner mitochondrial membrane (IMM) protein TIM23 (Figure 3A), suggesting that IFI27 is localized at the OMM but not the IMM. Moreover, when the specific transmembrane (TM) domain or mitochondrial targeting signal (MTS) was deleted, as shown in Figure 3B, none of the deletion mutants—ΔMTS, ΔTM1, ΔTM2, or ΔTM3 could be found in mitochondria (Figure 3C). Furthermore, none of them could promote the activation of GSDME (Figure 3D) or induce cell pyroptosis (Figure 3E), however, the WT‐IFI27 triggered marked cell pyroptosis along with GSDME activation (Figure 3D,E). Therefore, these findings demonstrate that the localization to mitochondria is required for IFI27 to induce cell pyroptosis.

### 3.4 IFI27 Impairs the Integrity and Function of Mitochondria

**FIGURE 4:**
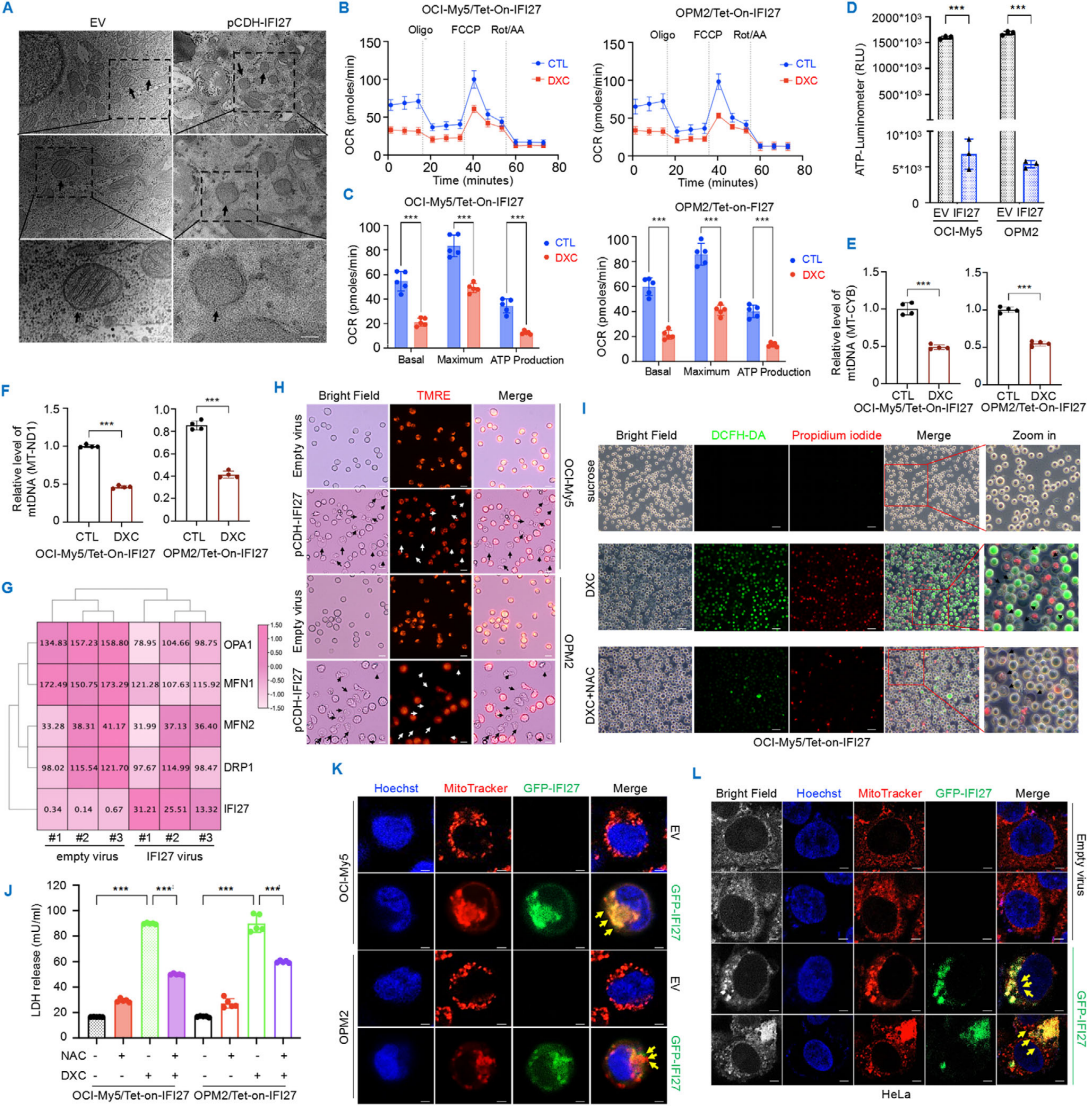
IFI27 impairs mitochondria and disrupts their integrity in MM cells. (A) HeLa cells were infected with lentiviral IFI27 for 72 h, followed by transmission electron microscopy (TEM) analysis. Arrows indicated mitochondria. Scale bar, 200 nm. (B, C) Tet‐on/inducible IFI27‐expressing MM cells were treated with doxycycline (DXC, 1 µg/mL) followed by measurement of the mitochondrial respiratory capacity using the Agilent Seahorse XF ATP Real‐Time rate assay with a Seahorse XFe96 analyzer (B). Data are analyzed statistically at least five independent experiments (C) (n = 5). D, OCI‐My5, and OPM2 cells were infected with lentiviral IFI27 for 72 h, followed by ATP measurement (n = 3). (E, F), Tet‐on/inducible IFI27‐expressing MM cells were treated with doxycycline (DXC), followed by qRT‐PCR to measure mtDNA levels using representative mitochondrial genes MT‐CYB (E) and MT‐ND1 (F). Data were analyzed statistically from at least four independent experiments (n = 4). (G) OCI‐My5 cells were infected with lentivirus IFI27 for 72 h; cells were then prepared for RNA‐seq, followed by a heatmap of genes related to mitochondrial dynamics. (H) MM cells were infected with lentiviral IFI27 for 72 h, followed by TMRE staining. Scale bar, 20 µm. (I, J) OCI‐My5 cell stably infected with an IFI27 (Tet‐on) lentiviral plasmid was treated with N‐acetyl cysteine (NAC, 5 mm) for 5 h. Cells were further treated with doxycycline (DXC) for 48 h, followed by DCFH‐DA or PI staining (I), and culture media were subjected to LDH release measurement (J) (n = 5). Scale bar, 50 µm. (K) OCI‐My5 and OPM2 cells were infected with lentiviral GFP‐IFI27 for 72 h, followed by MitoTracker staining and fluorescence microscopy analysis. Arrows indicated aggregated mitochondria. Scale bar, 5 µm. (L) HeLa cells were infected with lentiviral GFP‐IFI27 for 72 h, followed by MitoTracker staining and fluorescence microscopy analysis. Arrows indicated aggregated mitochondria. Scale bar, 10 µm. ^**^ *p* < 0.01; ^***^ *p* < 0.001. EV, empty virus.

To further explore the underlying molecular mechanism, we performed transcriptome sequencing after overexpressing IFI27 in myeloma cells. The GO analysis revealed that IFI27 overexpression effectively induced immune responses and upregulated of the type I interferon signaling pathway (Figure S1A). However, the downregulated genes were significantly enriched in biological processes related to mitochondrial structures (such as mitochondrial membrane and matrix) and metabolism (Figure S1B), so we hypothesize that IFI27 disrupts mitochondrial function. Given that IFI27 localization to mitochondria is important for its activity to induce pyroptosis (Figure 3) and mitochondria are at the center stage of cell pyroptosis [18], we wondered whether IFI27‐induced MM cell pyroptosis was associated with mitochondrial impairment. Transmission electron microscope (TEM) analyses showed that mitochondria became shrunken and swollen, and some mitochondrial membranes and cristae disappeared in cells infected with IFI27 lentivirus (Figure 4A). Subsequent Seahorse Cell Mito Stress Test Assays showed ectopic IFI27 decreased the basal oxygen consumption rate (OCR), the respiratory capacity, and the ATP production of mitochondria (Figure 4B–D). In addition, mitochondrial genes involved in oxidative phosphorylation, such as MT‐CYB and MT‐ND1, were markedly decreased (Figure 4E,F), but the associated genes in mitochondrial fusion and fission were not markedly altered (Figure 4G). These findings suggest that ectopic expression of IFI27 mainly impairs mitochondrial oxidative phosphorylation. Moreover, enforced IFI27 strikingly reduced mitochondrial membrane potential (Figure 4H and Figure S2A) and increased production of ROS (reactive oxygen species) (Figure S2B,C). Furthermore, ROS production and cell pyroptosis were partly abolished by NAC (N‐acetyl‐L‐cysteine), a typical antioxidant scavenger (Figure 4I and Figure S2D,E). Notably, IFI27‐promoted GSDME activation was also prevented by NAC (Figure 4J and Figure S2F). Furthermore, ectopic IFI27 led to mitochondrial aggregation in pyroptotic MM cells (Figure 4K), a hallmark of impaired mitochondria [19], which was also confirmed in HeLa cells (Figure 4L). Therefore, ectopic IFI27 impairs mitochondrial structure and function in association with pyroptosis.

### 3.5 Mitochondria are Required for IFI27‐Induced MM Cell Pyroptosis

**FIGURE 5:**
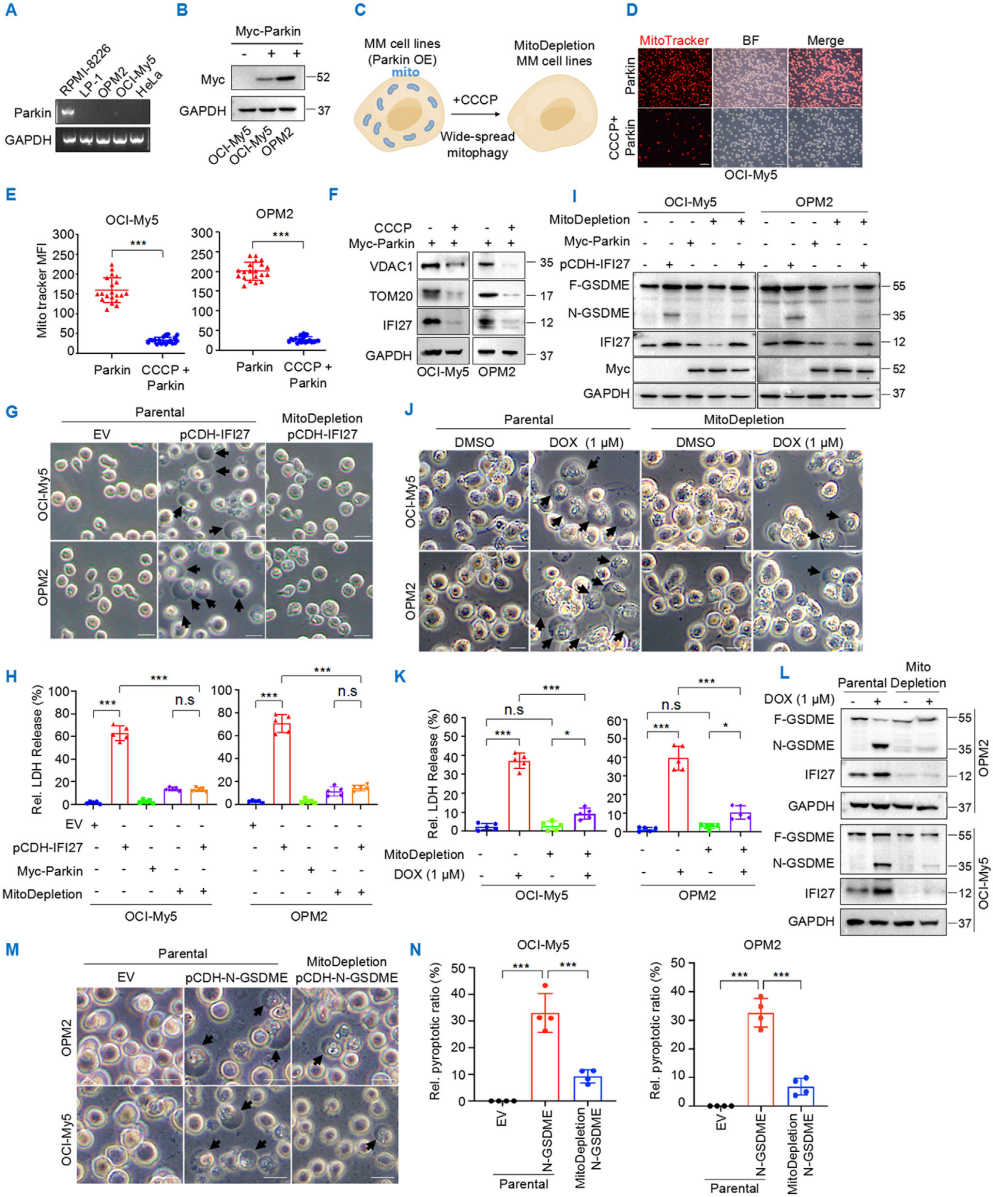
Mitochondria are required for IFI27‐induced MM cell pyroptosis. (A) Parkin expression in selected cell lines. (B) OCI‐My5 cells were infected with lentiviral Myc‐Parkin. (C) Schematic of the experimental approach. (D–F) Mitophagy was triggered in myeloma cells by expressing Parkin and treatment with CCCP (20 µm). Cells were stained with MitoTracker (D), statistical analysis of mean mitochondrial fluorescence intensity (MFI, E) (n = 20), and IB assays against TOM20, VDAC1, and IFI27 (F). Scale bar, 100 µm. (G–I) OPM2 and OCI‐My5 cells stably infected with lentiviral Myc‐Parkin were treated with 20 µm CCCP at 90%–100% confluency in complete medium for 3 cycles, and each cycle was 12 h. After 3 cycles of treatment with CCCP, cells were further infected with lentiviral IFI27 for 72 h. After pyroptosis morphology was determined by a phase‐contrast microscope (G), culture media were subjected to LDH measurement (H) (n = 5), and cell lysates were prepared for IB assays against GSDME and associated proteins (I). Scale bar, 20 µm. (J–L) Mitochondria were depleted by inducing mitophagy as described in the Methods section. The cells were further treated with DOX (1 µm) for 24 h, followed by phase‐contrast microscopy analysis (J), LDH measurement (K) (n = 5), and IB assays against GSDME and IFI27 (L). Scale bar, 20 µm. (M, N), OPM2 and OCI‐My5 cells with depleted mitochondria were infected with lentiviral N‐GSDME for 72 h, followed by phase‐contrast microscopy analysis (M) and statistical analysis of pyroptosis rate (N) (n = 4). Scale bar, 20 µm. ^*^ *p* < 0.05; ^***^ *p* < 0.001; n.s, not significant. EV, empty virus. Mito Depletion: Parkin expression + CCCP treatment to deplete mitochondria.

Given that IFI27 impairs mitochondria and disrupts their integrity in MM and HeLa cells, and mitochondria are a central node for cell death [18], we wondered whether mitochondria are required for IFI27 to induce cell pyroptosis. It is known that induction of mitophagy is an efficient manner to deplete mitochondria by treating Parkin‐expressing cells with CCCP, a mitochondrial uncoupler [13]. Because Parkin was not found in both OCI‐My5 or OPM2 cells (Figure 5A), these cells were infected with lentiviral Myc‐Parkin (Figure 5B), followed by exposure to CCCP to induce mitophagy (Figure 5C and Figure S3A) as monitored by MitoTracker staining and depletion of TOM20 and VDAC1, two mitochondrial marker proteins (Figure 5D–F and Figure S3A). Mitochondrial depletion was also evaluated by mitochondrial membrane potential (MMP) dissipation as TMRE staining (Figure S3B,C). Although CCCP also reduced MMP as TMRE staining (Figure S3B,C) and decreased MM cell viability (Figure S4A), CCCP did not induce marked cell plasma membrane rupture because PI‐positive cells were not strikingly increased as compared to the control (Figure S4C). In contrast, IFI27 strikingly decreased cell viability and induced PI positive cells (Figure S4B,C), however, when mitochondria were depleted, IFI27 almost failed to induce MM cell pyroptosis as measured by pyroptotic morphology (Figure 5G and Figure S5A) and PI staining (Figure S4C), LDH release (Figure 5H) and GSDME activation (Figure 5I). In this case, IFI27‐decreased cell viability was also partly prevented by mitochondrial depletion (Figure S4B). Given that DOX induces IFI27, which further triggers GSDME‐dependent MM cell pyroptosis, we next examined the effects of mitochondrial depletion on the role of both DOX and N‐GSDME in MM cell pyroptosis. Out of expectation, when mitochondria were depleted, neither DOX nor N‐GSDME induced MM cell pyroptosis (Figure 5J–N, and Figure S5B). Therefore, mitochondria are required for IFI27‐ and GSDME‐induced MM cell pyroptosis.

### 3.6 IFI27 Recruits N‐GSDME to Mitochondria for Triggering MM Cell Pyroptosis

**FIGURE 6:**
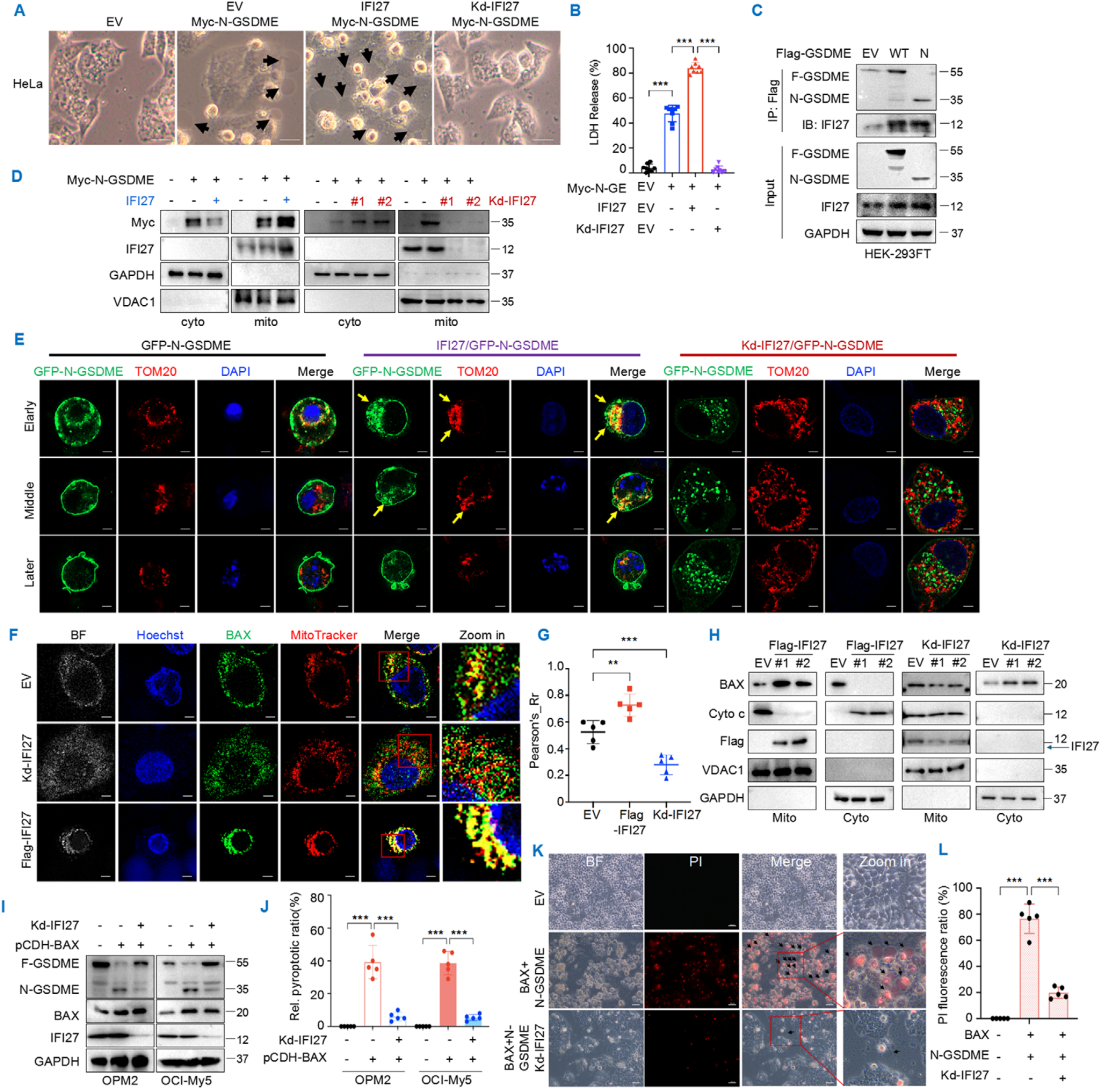
IFI27 recruits N‐GSDME to mitochondria to trigger MM cell pyroptosis. (A, B), HeLa cells were subjected to IFI27 overexpression or knockout followed by transfection with an N‐GSDME plasmid for 48 h. Cells were then subjected to phase‐contrast microscopy (A) and LDH measurement from the culture media (B) (n = 8). Scale bars, 50 µm. (C), HEK‐293FT cells were transfected with WT‐GSDME or N‐GSDME for 48 h, and cell lysates were subjected to Co‐IP assay. (D), HeLa cells were infected with the indicated lentivirus for 72 h, followed by isolation of the mitochondrial cytosol fractions and IB assay as indicated. (E), HeLa cells stably expressing IFI27 or with IFI27 knockdown were transfected with GFP‐N‐GSDME, followed by TOM20 and DAPI staining before being analyzed by confocal microscope analysis (E). Scale bars, 10 µm. (F–H), HeLa cells were subjected to IFI27 overexpression or knockdown for 72 h, followed by staining with Hoechst, BAX, and MitoTracker before being analyzed on a confocal microscope (F), the Pearson analysis of association of BAX and mitochondria (G) (n = 5), and measurement of the distribution of BAX and cyto c in the mitochondria (Mito) and cytosol (Cyto) fractions (H). Scale bars, 10 µm. (I, J), MM cells were infected with lentivirus to knockdown fIFI27 as indicated; cells were then infected with BAX lentiviral before being subjected to IB assays (I), and the proportion of pyroptotic cells was analyzed (J) (n = 5). (K, L), After infection with lentivirus to knock down IFI27 for 72 h, HeLa cells were transfected with Myc‐N‐GSDME and BAX plasmids, followed by PI staining (K), the PI‐positive cells ratio was analyzed (L) (n = 5). Scale bars, 50 µm. ^*^ *p* < 0.05; ^**^ *p* < 0.01; ^***^ *p* < 0.001. EV, empty virus.

Given IFI27 mediates MM cell pyroptosis in a GSDME‐dependent manner (Figure 2), we wondered about the underlying mechanism. As expected, IFI27 strikingly enhanced N‐GSDME‐mediated cell pyroptosis (Figure 6A,B), but it was ablated by the knockout of IFI27 (Figure 6A,B), suggesting N‐GSDME requires IFI27 for cell pyroptosis. Given that N‐GSDME should be translocalized to mitochondria before pyroptosis, we next evaluated the effects of IFI27 on N‐GSDME translocalization to mitochondria. As shown in Figure 6C, IFI27 bound to the N‐terminus of GSDME (Figure 6C) and promoted its distribution to mitochondria (Figure 6D). Consistent with this finding, IFI27 knockout prevented N‐GSDME translocalization to mitochondria (Figure 6D), which was further confirmed by immunofluorescence staining and confocal microscopy analyses (Figure 6E). Overexpression of IFI27 strikingly increased the distribution of N‐GSDME in both mitochondria and the plasma membrane at the early stage, but at the later stage of pyroptosis, N‐GSDME was mainly found in the plasma membrane (Figure 6E). However, when IFI27 was knocked down (Kd), no GSDME was found in mitochondria or the plasma membrane either (Figure 6E), further suggesting IFI27 recruits N‐GSDME to mitochondria before triggering pyroptosis. Moreover, given BAX also promotes MM cell pyroptosis via GSDME [4], we next evaluated the effects of IFI27 in BAX‐mediated cell pyroptosis. As shown in Figure 6F–H, BAX could be seen in mitochondria, and it was markedly increased by IFI27 but almost abolished by knockdown of IFI27 (Figure 6F–H). Consistent with this finding, ectopic IFI27 promoted BAX to mitochondria, which further led to mitochondrial membrane permeabilization and cyto c markedly increased in the cytosol (Figure 6H). In contrast, knockdown of IFI27 abolished BAX in mitochondria, and cyto c in the cytosol was reduced (Figure 6H). This finding was also confirmed in BAX‐induced cell pyroptosis. Knockdown of IFI27 markedly ablated BAX‐induced MM cell pyroptosis, as evidenced by GSDME activation (Figure 6I) and morphological alteration (Figure 6J). Furthermore, IFI27 knockdown prevented BAX/N‐GSDME‐induced cell pyroptosis (Figure 6K,L). IFI27 knockout also almost ablated DOX‐induced MM cell pyroptosis (Figure S6A,B). Collectively, these results indicate that N‐GSDME must be recruited to mitochondria by IFI27 before initiating MM cell pyroptosis.

### 3.7 IFI27 Requires BAX to Trigger MM Cell Pyroptosis

**FIGURE 7:**
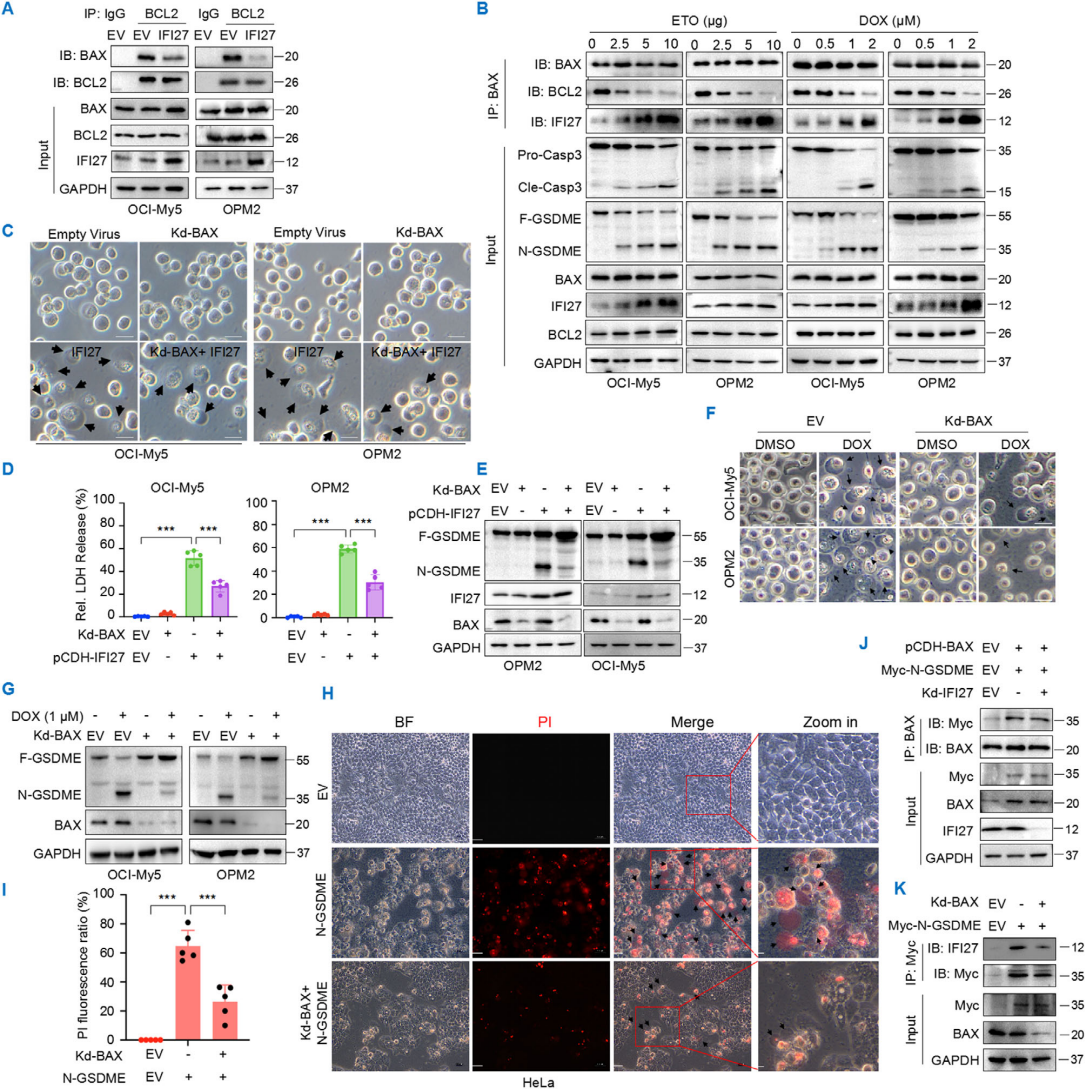
IFI27 requires BAX to trigger MM cell pyroptosis. (A) MM cells were infected with IFI27 lentivirus for 72 h, cell lysates were prepared for IP/IB assays as indicated. (B) Myeloma cells were treated with etoposide (ETO) or doxorubicin (DOX) for 24 h, and cell lysates were then prepared for IP/IB assays as indicated. (C–E) OPM2 and OCI‐My5 cells were knocked down for BAX, followed by infection with lentiviral IFI27 for 72 h. Cells were then subjected to phase‐contrast microscopy analyses (C), culture media were subjected to LDH measurement (D) (n = 5), and cell lysates were subjected to IB assays (E). Scale bar, 20 µm. (F, G) Myeloma cells were knocked down for BAX for 72 h, followed by DOX treatment for another 24 h before being subjected to phase‐contrast microscopy (F), cell lysates were prepared for IB assays (G). Scale bar, 20 µm. (H, I) HeLa cells were infected with Kd‐BAX lentivirus, followed by transfection with an N‐GSDME plasmid for 48 h, followed by PI staining (H) and statistical analysis on PI‐positive cells (I) (n = 5). Scale bar, 50 µm. (J) After infection with lentivirus to knock down IFI27 for 72 h, HeLa cells were transfected with Myc‐N‐GSDME and BAX plasmids, followed by IP/IB assays as indicated. (K) HeLa cells were infected with lentivirus to knock down BAX for 72 h before being transfected with the Myc‐N‐GSDME plasmid, followed by IP/IB assays. ^***^ *p* < 0.001. EV, empty virus.

The above study demonstrated that IFI27 triggers mitochondria‐dependent pyroptosis; however, IFI27 is a mitochondrial protein, but in normal condition it fails to induce MM cell pyroptosis and cell death, which suggests that in response to pyroptosis signals, IFI27 is upregulated and further requires adaptor proteins to impair mitochondria before inducing cell pyroptosis. It is known that mitochondrial integrity is well maintained by the Bcl‐2 family proteins, such as Bcl‐2/BAX, and BAX binds to N‐GSDME [5]. We therefore wondered whether IFI27 affects Bcl‐2/BAX homeostasis. The Co‐IP assay showed that ectopic IFI27 markedly decreased the interaction between Bcl‐2 and BAX (Figure 7A). Moreover, both DOX and ETO promoted the expression of IFI27 and disrupted the interaction between Bcl‐2 and BAX (Figure 7B). Meanwhile, the binding between BAX and IFI27 became stronger upon the increase of drug concentrations (Figure 7B), which suggests IFI27 might liberate BAX, and BAX further induces IFI27‐triggered MM cell pyroptosis. To validate this hypothesis, MM cells with BAX knockdown (Kd) were infected with lentiviral IFI27. The results showed that Kd‐BAX robustly ablated IFI27‐triggered GSDME activation and cell pyroptosis (Figure 7C–E). Furthermore, given that IFI27 is required for DOX and N‐GSDME‐mediated MM cell pyroptosis, we also examined the effects of BAX on these two pyroptosis triggers. It turned out that BAX knockdown significantly abolished DOX‐ and N‐GSDME‐induced pyroptosis (Figure 7F–I). Previous studies have demonstrated that BAX binds to N‐GSDME [5] and IFI27 binds to both BAX and N‐GSDME; therefore, these findings suggest that there is a close modulation between IFI27, BAX, and N‐GSDME. To determine this inter‐modulation, we knocked down BAX or IFI27; the subsequent assays showed that knockdown of IFI27 did not reduce the interaction between BAX and N‐GSDME (Figure 7J), but BAX knockdown markedly ablated the binding of IFI27 to N‐GSDME (Figure 7K). These results demonstrated that BAX might be an adapter or a mediator between IFI27 and N‐GSDME. BAX is required for IFI27 to induce GSDME‐dependent MM cell pyroptosis.

### 3.8 Ectopic IFI27 Reduces MM Tumor Burden and Increases the Overall Survival (OS) of Mice Bearing Myeloma via Inducing Pyroptosis

**FIGURE 8:**
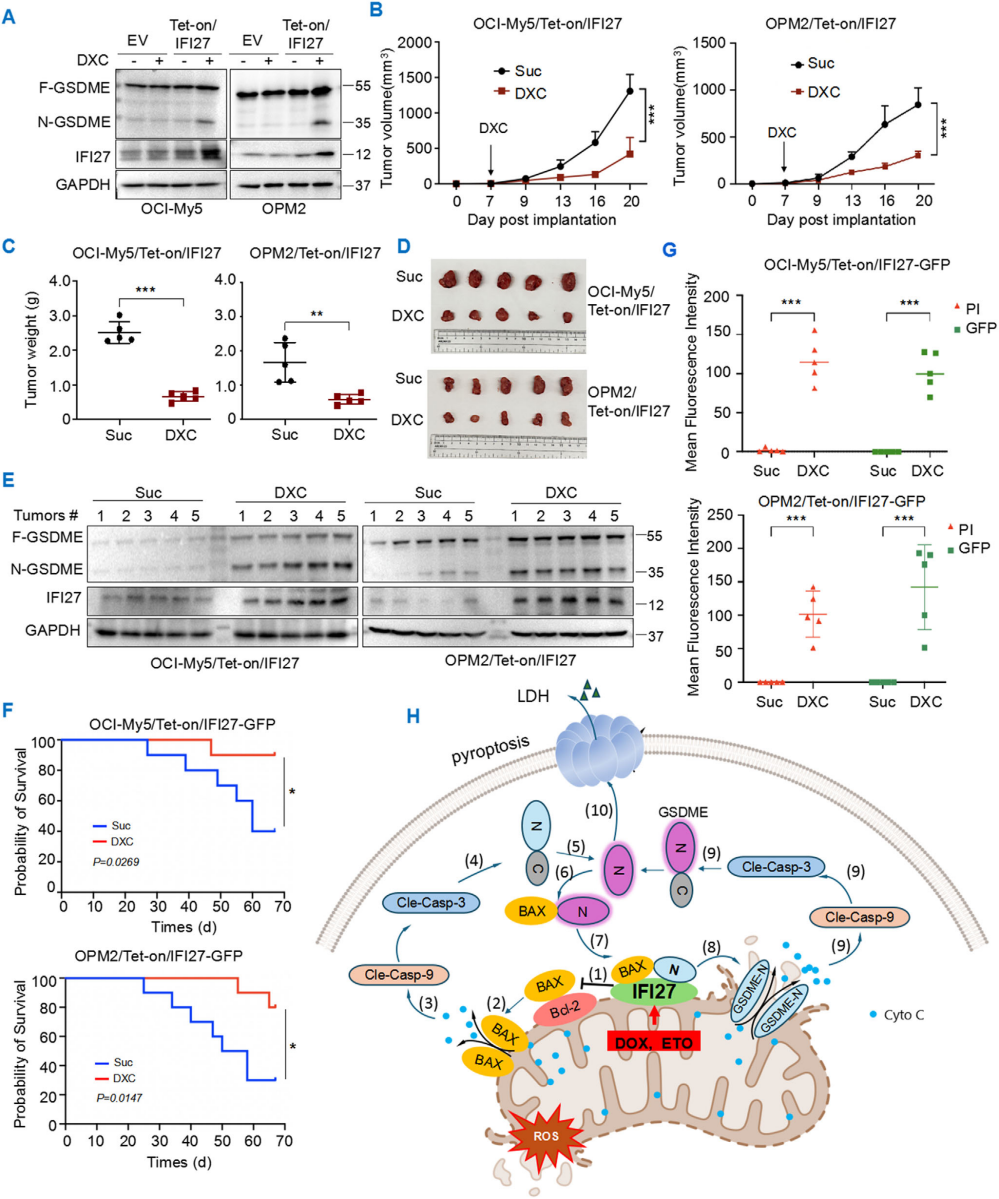
Ectopic IFI27 reduces MM tumor burden and prolongs the overall survival (OS) of myeloma‐bearing mice via the induction of pyroptosis. (A) OCI‐My5 and OPM2 cells stably infected with tetracycline‐inducible (Tet‐on) IFI27 lentivirus were treated with doxycycline (DXC) for 48 h, followed by IB assay. (B–D) OCI‐My5 and OPM2 cells with tet‐on/IFI27 were subcutaneously implanted into nude mice. Once tumors were palpable, mice were randomly divided into two groups; each was treated with DXC or sucrose (suc) for two weeks. Tumor growth rate (B), tumor weight (C), and tumor image (D) were detected as indicated (n = 5). (E) Tumor tissues were subjected to IB for the indicated proteins. (F, G) OCI‐My5 and OPM2 cells were stably infected with lentiviral Tet‐on/IFI27‐GFP. Cells were injected into nude mice via the tail vein. Seven days later, mice were treated with DXC for 5 d per week. Mice survival was then monitored (F). At the end of the experiment, bone marrow cells were collected and stained with PI, followed by mean fluorescence intensity analysis (G) (n = 5). ^*^ *p* < 0.05; ^**^ *p* < 0.01; ^***^ *p* < 0.001. (H) The proposed model of GSDME‐mediated MM cell pyroptosis that is modulated by IFI27 and BAX. IFI27 is downregulated in MM cells. Upon pyroptotic stimulation, such as DOX or ETO treatment, IFI27 is induced, therefore liberating BAX from BCL‐2 (1). BAX is then recruited to mitochondria and forms homo‐oligomeric channels in OMM, therefore leading to cytochrome C leaks to the cytosol (2) and activating Caspase‐3 (3), which further cleaves and activates GSDME (4 and 5). The resultant N‐GSDME is further identified by BAX (6) and is recruited to OMM by IFI27 (7). N‐GSDME forms pores in the outer membrane of mitochondria (OMM) and also leads to cytochrome C release (8), therefore further activating Caspase‐9/Caspase‐3/GSDME (9). More N‐GSDME fragments then form pores in the plasma membrane, leading to cell pyroptosis (10). The upregulated IFI27, liberated BAX, and activated GSDME collaborate in mitochondrial structural damage and dysfunction that further exaggerates MM cell pyroptosis.

Given that overexpression of IFI27 can induce robust MM cell pyroptosis in vitro, we wondered its anti‐MM activity in vivo. To this end, we constructed a Tet‐on‐IFI27 lentiviral system in both OCI‐My5 and OPM2 cells (Figure 8A), which were further injected subcutaneously into immunodeficient mice. When tumors were palpable, mice were randomly grouped and administered sucrose solution with or without 3.2 mg/mL doxycycline (DXC), a promoter of the tet‐on‐inducible genes [16]. The results showed that DXC strikingly reduced MM xenograft growth in both models (Figure 8B–D). As expected, DXC strikingly induced IFI27 expression associated with GSDME activation (Figure 8E). Moreover, DXC‐induced IFI27 expression significantly increased OS in both models (Figure 8F) in association with a marked increase in the percentage of GFP^+^ (IFI27 fusion) and PI ^+^ (pyroptosis hallmark) cells (Figure 8G). Therefore, these in vivo studies demonstrated that induction of IFI27 not only inhibits the growth of MM xenografts but also prolongs the survival of mice bearing MM tumors.

## 4 Discussion

The above studies demonstrate that the mitochondrial resident protein IFI27 induces MM cell pyroptosis in a mitochondria‐ and GSDME‐dependent manner by partnering with BAX. Upon pyroptotic stimuli, upregulated IFI27 binds to and recruits BAX and N‐GSDME to mitochondria, therefore impairing mitochondrial integrity and function, increasing mitochondrial permeability, and finally leading to MM cell pyroptosis. Both IFI27 and BAX are essential for mitochondria‐dependent and GSDME‐mediated pyroptosis.

IFNs have been widely used in the treatment of viral infections and some cancers. In the treatment of MM, IFNs show great clinical benefit, including but not limited to increased induction rate and prolonged overall survival [8]. The mechanisms might be associated with upregulated targeted genes (ISGs) via the JAK1/STAT1, IRF3, PI3K, and MAPKp38 signaling pathways [20]. However, each ISG may act different functions; for example, IFIT1 and IFIT3 participate in MM cell pyroptosis but are unable to induce pyroptosis [5, 6]. The present study finds that, different from IFIT1 and IFIT3, IFI27 is capable of triggering MM cell pyroptosis by activating GSDME. This finding suggests that although IFNs can upregulate the expression of hundreds of genes [20], only a small subset of ISGs displays pyroptosis‐inducing activity. This is probably explained by the finding that expanded expression of atypical ISGs drives cancer cell pyroptosis [21]. Moreover, individual ISGs may use their own specific pyroptotic executors; for example, IFI16 induces CD4^+^ T cell pyroptosis by activating GSDMD [22], while IFI27 triggers MM cell pyroptosis via activating GSDME but no other GSDMs (Figure 2). Moreover, IFI27 is able to induce MM cell pyroptosis by recruiting BAX and GSDME to mitochondria and impairing mitochondrial structure and function.

Mitochondria are membrane‐bound organelles that not only the ATP‐producing plant but also a commander that determines cell fate. In addition to apoptosis, ferroptosis, and curoptosis, mitochondria also play an important role in pyroptosis. In the present study, we find that mitochondria are required for MM cell pyroptosis induced by BAX, GSDME, and IFI27. However, mitochondrial damage may not induce pyroptosis. CCCP is a mitochondrial uncoupler [13], although its prolonged treatment can damage mitochondria and lead to apoptosis and mitophage but not marked pyroptosis as assayed by our PI staining and morphology analysis, suggesting that CCCP treatment can't result in plasma membrane rupture. This finding is consistent with a previous study in which CCCP does not induce marked melanoma cell pyroptosis without co‐treatment with iron such as ferrous sulfate [13]. Moreover, CCCP/Parkin‐induced mitochondrial depletion via mitophagy does not induce pyroptosis, either. In the absence of mitochondria, we find that neither N‐GSDME, BAX, DOX, nor IFI27 can induce MM cell pyroptosis because mitochondria are depleted. This holds true because when mitochondria are depleted, mitochondria‐associated pyroptotic signals can't be activated. Moreover, N‐GSDME should be translocated to mitochondria before it moves to the plasma membrane in a similar manner to GSDMD [13]. However, how N‐GSDME is translocated from mitochondria to the plasma membrane before triggering cell pyroptosis remains a very interesting issue to be investigated.

Although there are five known pyroptosis executors, IFI27‐triggered MM cell pyroptosis relies on GSDME but not GSDMD (Figure 2), which contradicts IFI16, which induces CD4 T cell pyroptosis via GSDMD [22], but is consistent with MM or other cancer cell pyroptosis induced by DOX [9] or BAX [4] or FOXO3 [16]. These findings suggest that the modulation of GSDMD and GSDME might be different. GSDMD is the major executor in macrophage cell pyroptosis; moreover, GSDMD is translocalized to mitochondria via binding to the externalized cardiolipin but independent of BAX or BAK [7]. However, in terms of GSDME, we find it relies on BAX and IFI27. In the absence of BAX or IFI27, GSDME can't be translocalized to mitochondria. We believe that, compared to GSDMD, which recognizes and binds to externalized cardiolipin, it might be more efficient for GSDME translocalization to mitochondria driven by IFI27 because IFI27 is a mitochondrial resident protein.

Moreover, IFI27 affects BAX translocation, cytochrome c release, and GSDME cleavage (Figures 2 and 6), which naturally places this pathway at the interface between mitochondrial apoptosis and GSDME‐driven pyroptosis. Previous studies have demonstrated that both ectopic IFI27 and BAX are reported to induce cell apoptosis in association with mitochondrial dysfunction [11, 23]. We believe GSDME, the pyroptotic executor, makes the fate decision on cell pyroptosis induced by BAX or IFI27, because when GSDME is knocked out, neither IFI27 (Figure 2) nor BAX [4] can induce MM cell pyroptosis. Moreover, IFI27 and BAX are mutually important for their action in MM cell pyroptosis. When IFI27 is knocked down, pyroptosis induced by BAX, chemotherapeutic agents, and N‐GSDME is significantly reduced (Figure 6). On the other hand, when BAX is knocked out, IFI27 will not be able to induce cell pyroptosis (Figure 7). Compared with GSDMD that solely relies on externalized cardiolipin, the modulation of GSDME associated with mitochondria is more complicated and fine‐tuned. All these findings demonstrate that IFI27 and BAX are interdependent and both are essential for mitochondria‐ and GSDME‐dependent pyroptosis.

Therefore are 300–500 ISGs, and as reported, specific ISGs may display their own specific biological activity in different contexts and different cell types. In terms of IFI27, it also plays a dual role, typically supporting immune defense but sometimes contributing to disease progression [24]. It is reported that IFI27 may act as an oncogene in B‐cell lymphoma and esophageal squamous cell carcinoma [25]. But it shows great activity against breast and ovarian cancers [26] and induces cell apoptosis by inhibiting Bcl‐2 activity [27]. In the present study, we find IFI27 is a pro‐pyroptosis gene against MM. IFI27 is downregulated in MM cells, but it is upregulated by chemotherapeutic agents. Moreover, induction of IFI27 induces MM cell pyroptosis, delays growth of MM xenografts, and therefore prolongs the OS of mice bearing MM. Interestingly, GSDME is also downregulated in MM cells, and its low expression indicates an inferior outcome [16]. Moreover, induction of GSDME by FOXO3 and the natural product corylin leads to delayed MM xenograft growth [16]. Our unpublished data also find that IFI27 can upregulate GSDME. These findings suggest that IFI27 could be developed as a therapeutic target against MM.

All in all, the present study reveals a novel modulation in GSDME‐mediated pyroptosis using the mitochondrial IFI27 as the central platform. IFI27 is downregulated in MM cells. Upon pyroptotic stimulation, such as DOX or ETO treatment, IFI27 is induced, therefore liberating BAX from BCL‐2. BAX is then recruited to mitochondria and forms homo‐oligomeric channels in OMM, therefore leading to cytochrome C leaks to the cytosol and activating Caspase‐3, which further cleaves and activates GSDME. The resultant N‐GSDME is further identified by BAX and therefore is recruited to OMM by IFI27. Upregulated IFI27, liberated BAX, and activated GSDME trigger mitochondrial structural damage and dysfunction that further exaggerates pyroptosis (Figure 8H). Tumor cells such as MM cells are then triggered to GSDME‐mediated pyroptosis, and tumor growth is suppressed. IFI27 and BAX duo are essential for mitochondria‐derived and GSDME‐dependent MM cell pyroptosis. Induction of IFI27 is also a novel promising venue for MM treatment via induction of pyroptosis.

## Author Contributions

X.M. and L.X. designed the study; Y.C., Y.S., Z.L., Y.Z., Y.W., and Y.H. conducted experiments; X.M. and Y.C. analyzed data; D.F., F.X., X.W., and L.X. provided key materials. Y.C., L.X., and X.M. wrote the manuscript.

## Funding

This study was partly supported by the National Natural Science Foundation of China (Nos. 82670289, 82170176 to X.M., 82470201 to L.X.), and by the Scientific Research Foundation of Shandong Province of Outstanding Young Scientists (ZR2025QA21).

## Conflicts of Interest

The authors declare no conflicts of interest.

## Supporting information




**Supporting File**: advs77164‐sup‐0001‐SuppMat.docx.

## Data Availability

The data that support the findings of this study are available from the corresponding author upon reasonable request.
