# Supplementary material for: IFI27 and BAX are Essential for GSDME‐Mediated Myeloma Cell Pyroptosis
Source: Adv Sci (Weinh). 2026 Aug 30:e77164. Online ahead of print. doi: 10.1002/advs.77164 (PMC13526420; doi:10.1002/advs.77164)
Supplement: Supplementary file 1 [file ADVS-9999-e77164-s001.docx]

**The Supplemental materials for the submission:**

**IFI27 and BAX are essential for GSDME-mediated myeloma cell pyroptosis**

Yaoli Cui^1^, Yuening Sun^1^, Ziyang Liu^1^, Xuntao Lai^1^, Yunshu Zhao^1^, Yaner Wang^1^, Yuanming He^1^, Fang Xu^2^, Du Feng^2^, Longlong Liu^1^, Zhenqian Huang^1^, Lijie Xing^3*^, Xinliang Mao^1, *^

^1^ Department of Hematology, The Key Laboratory of Advanced Interdisciplinary Studies, The First Affiliated Hospital of Guangzhou Medical University; Guangdong Provincial Key Laboratory of Protein Modification and Degradation, School of Basic Medical Sciences, Guangzhou Medical University, Guangzhou 511436, P. R. China

^2^ Department of Pathophysiology, School of Basic Medical Sciences, Guangzhou Medical University, Guangzhou 511436, P. R. China

^3^ Department of Lymphoma, Shandong Cancer Hospital and Institute, Shandong First Medical University and Shandong Academy of Medical Sciences, Jinan, Shandong, China.

***Materials and Methods***

***Cell culture***

RPMI-8226, OCI-My5, OPM2, HEK-293FT, and HeLa cell lines were obtained from American Tissue and Cell Collection (ATCC, https://www.atcc.org) or generously provided by Dr. Aaron Schimmer, University of Toronto, Canada. MM cells were cultured in Iscove’s modified Dulbecco’s medium (IMDM). HEK-293FT and HeLa cells were maintained in Dulbecco’s modified Eagle’s medium (DMEM). All cells were grown at 37°C in a 5% CO_2_ incubator. Bone marrow species were voluntarily donated by MM patients with a written content in accordance with the the Helsinki Declaration principles and this study was approved by the Medical Ethics Board of the First Affiliated Hospital of Guangzhou Medical University (Approval No. #ES-2024-201-01). All media were supplemented with 10% FBS (Cat. #F103, Vazyme Biotech Co., Ltd, Nanjing, China) and 1% penicillin/streptomycin (Cat. #P4458, Sigma-Aldrich).

***Antibodies and reagents***

The antibodies used for co-immunoprecipitation assays, western blot assays or immunofluorescence analyses were as follows: Anti-IFI27 (Cat. #45633) was from Signalway Antibody LLC (Greenbelt, MD). Anti-caspase-1 (Cat. #2225S) was from Cell Signaling Technology (Boston, MA). Anti-caspase-3 (Cat. #19677-1-AP), anti-caspase-9 (Cat. #10380-1-AP), anti-BAX (Cat. #50599-2-Ig), anti-Bcl-2 (Cat. #60178-1-Ig), anti-TOM20 (Cat. #11802-1-AP), anti-cytochrome c (Cyt c, Cat. #10993-1-AP), anti-VDAC1 (Cat. #55259-1-AP), and anti-GAPDH (Cat. #10494-1-AP) were from Proteintech (Wuhan, China). Anti-DFNA5/GSDME (Cat. #ab215191) and anti-GSDMB (Cat. #ab215729) were obtained from Abcam Inc. (Waltham, MA). Anti-GSDMA (Cat. #PA5-24813) and Anti-GSDMC (Cat. #PA5-24259) were from Invitrogen. Anti-GSDMD (Cat. #NBP2-33422) was from Novus Biologicals (Centennial,CO). BeyoWB™ HRP Labeled Goat Anti-Rabbit IgG (H+L) (Cat. #P0948), BeyoWB™ HRP Labeled Goat Anti-Mouse IgG (H+L) (Cat. #P0946), Alexa Fluor 488 Goat Anti-Rabbit (Cat. #A0423), Alexa Fluor 488 Goat Anti-Mouse (Cat. #A0428), Alexa Fluor 594 donkey anti-Rabbit (Cat. #A1152), Alexa Fluor 594 donkey anti-Mouse (Cat. #A1143), Mouse IgG (Cat. #A7028) and Rabbit IgG (Cat. #A7016) were purchased from Beyotime Institute of Biotechnology (Shanghai, China). Etoposide (ETO) (Cat. #S1225), cisplatin (CDDP) (Cat. #S1166), bortezomib (BZ) (Cat. #S1013), clioquinol (CLQ) (Cat. #S4601), and doxorubicin (DOX) (Cat. #E2516) were obtained from Selleck Chemicals (Shanghai, China). Doxycycline (DXC) (Cat. #ST039A), Hoechst 33342 (Cat. #C1026), DAPI (Cat. # C1002), propidium iodide (Cat. #ST511), lipopolysaccharide (LPS, Cat. #ST1470), protease inhibitor cocktail (Cat. #P1005) and phenylmethanesulfonyl fluoride (PMSF) (Cat. #ST505) were purchased from Beyotime. Mito Tracker Red CMX Ros (Cat. #M7512) was purchased from Invitrogen, Polo Alto, CA. IP lysis buffer was from X-Blot Biotechnology (Suzhou, China) (Cat. #Xblot-012).

***Primers for plasmid constructs***

pCDH-IFI27: 5’-GGAATTCATGGACTACAAGGATGACGATGACAAGGAGGC CTCTGCTCTCA-3’ (forward) and 5’-CGGGATCCCTAGTAGAACCTCGCAAT GACA-3’ (reverse).

pCDH-BAX: 5’-CGGAATTCATGGACTACAAGGATGACGATGACAAGGACGG GTCCGGGGAGCAGCCCA-3’ (forward) and 5’-ATAAGAATGCGGCCGCTTAT GGAGGAAAAACACAGTCCA -3’ (reverse).

pCDH-N-GSDME: 5’-CGGAATTCATGGACTACAAGGATGACGATGACAAGTT TGCCAAAGCAACCAGGA-3’ (forward) and 5’-CGGGATCCATCTGGCATGTC TATGAATG-3’ (reverse).

Myc-N-GSDME: 5’-CTTGGTACCGAGCTCGGATCCATGTTTGCCAAAGCAAC CAGG-3’ (forward) and 5’-AACGGGCCCTCTAGACTCGAGTCAATCTGGCAT GTCTATGAATGC-3’ (reverse).

mCherry-IFI27 (ΔMTS):

5’-ATGACAAGGCTACAGTTGTGATTGGAGG-3’ (forward) and

5’-ACTGTAGCCTTGTCATCGTCATCCTTGT-3’ (reverse).

mCherry-IFI27 (ΔTM1):

5’-CCAGGATTGCGGCGGGAATCGCCTCGTC-3’ (forward) and

5’-CCCGCCGCAATCCTGGCCAGGGGCAAA-3’ (reverse).

mCherry-IFI27 (ΔTM2):

5’-CCAAGATGCAGTCACTGGGAGCAACTGG-3’ (forward) and

5’-AGTGACTG CATCTTGGCTGCTATGGAGG-3’ (reverse).

mCherry-IFI27 (ΔTM3):

5’-CAACTGGAAGGTTCTACGCGGATCCACC-3’ (forward) and

5’-TAGAACCT TCCAGTTGCTCCCAGTGACT-3’ (reverse).

***CRISPR-Cas9 knockout (KO) and the lentiviral system***

The CRISPR design tools at Benchling (https://benchling.com) were used to identify single guide (sg) RNAs. The oligonucleotides were annealed and subcloned into the lentiviral vector LentiCRISPR v2-Puro (Addgene.org). The lentivirus was generated by transfecting HEK293T cells with the lentiviral vector and packaging plasmids. The viral supernatants were collected after 48 h and then infected into cells cultured in IMDM (Cat. #HY-K1057, MedChemExpress, Shanghai, China). After infecting lentivirus for 48 h, positive MM cells were selected with puromycin (500 ng/μL). The knockdown efficiency of the target genes was determined by Western blotting or RT-PCR.

***RT-PCR and primers***

Total RNA was extracted using a total RNA isolation kit (Beyotime, Shanghai, China). RNA was reverse-transcribed using EasyScipt® First-strand cDNA Synthesis SuperMix (TransGen Biotech Co., Ltd., Beijing, China) according to the manufacturer’s instructions. PCR amplification was performed using the following primers: IFI27 (forward): 5’-CTCTGGCTCTGCCGTAGTTT-3’; Reverse: 5’-ATGGAGGACGAGGCGATT-3’. GAPDH (forward): 5’-AGTCCACT GGCGTCTTCA; Reverse: 5’-CTCCGACGCCTGCTTCACCA-3’. Parkin (forward): 5’-CGATGCCACGACAATAGA-3’; Reverse: 5’-GGTTATATTCAGACGCTCCT- 3’. MT-ND1 (forward): 5’-CGATTCCGCTACGACCAACT; Reverse: 5’-AGGTTTGAGGGGGAATGCTG-3’. MT-CYB: 5’-CCCCACCCCATCCAACAT-3’; Reverse: 5’-TCAGGCAGGCGCCAAG-3’. The PCR products were analyzed by fluorescence quantitative analysis or visualized by GoldView® staining (TransGen) after electrophoresis on 2% agarose gels.

***RNA sequencing***

Cells of interest were plated into the 10-cm dishes (10^7^ cells/dish). One group was treated with DMSO, while the other group was stimulated by 2 μM of doxorubicin (DOX) for 24 h. Total RNAs from cells of interest were extracted with an RNA isolation kit as shown previously^1^. The prepared RNA samples (n = 3) were subjected to transcriptomic sequencing by IGE Biotechnology LTD (Guangzhou, China).

***Western blotting (WB)***

The cells were harvested and lysed using a lysis buffer contianing 1% NP-40 for 40 min on ice, and the resulting mixture was centrifuged at 12000 g for 20 min at 4°C. The liquid fraction (supernatant) was collected, and the total protein concentration was determined using the BCA Protein Quantification Kit (Cat. #P0009, Beyotime). All samples were standardized and heated at 100°C for 10 min. Samples and protein markers (Cat. #MP102-01, Vazyme) were separated by sodium dodecyl sulfate-polyacrylamide gel electrophoresis (SDS-PAGE) and transferred to polyvinylidene difluoride (PVDF) membranes (Millipore®). Membranes containing protein samples were blocked with 5% fat-free milk for 1 h at room temperature, after which they were incubated with primary antibodies overnight at 4°C^1^. After washing three times for 10 min each with TBST buffer (137 mM NaCl, 20 mM Tris, 0.1% Tween 20, pH = 7.5), the cells were incubated with secondary antibodies for 1 h at room temperature. Subsequently, after three additional washes with TBST buffer for 10 min each, the blots were visualized using High-sigECL Western blotting substrate (Cat. #E423, Vazyme) and Automatic chemiluminescence instrument (Cat. #5200SF, Tanon).

***Co-Immunoprecipitation (Co-IP)***

The IP assay was performed as described previously^2^. For direct co-immunoprecipitation of intracellular proteins, the treated cells were cultured in 10 cm dishes until they reached 80–90% confluence. The cells were then lysed using IP lysis buffer (Cat. #Xblot-012, X-Blot Biotechnology) for 40 min on ice. The lysate was centrifuged at 12000 × g for 30 min at 4°C, and the resulting supernatant was collected. A BCA kit was used to detect the total protein content in the cell supernatant. Approximately 3000 μg of protein was transferred to fresh 1.5-mL Eppendorf tubes and supplemented with specific target antibodies. The mixture was then incubated for 12 hours at 4°C. Subsequently, for immunoprecipitation, protein A + G agarose beads (Cat. #P2055; Beyotime) were prewashed three times with PBST (phosphate-buffered saline (PBS) containing 0.5% Tween-20) and added to the tubes for incubation for 4 hours at 4°C. They were then washed five times on ice with buffer (10 mM Tris, 150 mM NaCl, 1% NP-40, 0.1% SDS, and 0.1% deoxycholate (pH 7.4). Finally, the precipitated beads were boiled for 10 min with 2 × SDS loading buffer (3.3 g Tris-Hcl, 10 g SDS, 30 mL glycerinum，7.71 g DTT and 0.04 g bromophenol blue dissolved and fixed in 100mL double distilled water, pH = 7.5) for WB.

***Cell transfection***

Logarithmically growing cells were selected and seeded uniformly into cell culture dishes 24 h prior to transfection. Subsequently, the old culture medium was aspirated and discarded, and 10 mL of serum‑free medium was slowly added along the inner wall of the dishes. Transfection complexes were prepared and incubated at room temperature for 15-20 min. The complexes were then added dropwise and evenly to 10 cm cell culture dishes, followed by gentle shaking to ensure uniform distribution, after which the dishes were returned to the incubator for further culture. After 12 h, the serum‑free medium containing transfection reagent was carefully removed, and 12 mL of fresh complete medium (supplemented with 10% FBS and devoid of antibiotics) was added. Finally, cells were cultured until the designated time point for subsequent experimental assays.

***Lactate dehydrogenase (LDH) release assay***

LDH in culture media was measured by using the LDH Cytotoxicity Assay Kit (Cat. #C0017, Beyotime) following the manufacturer’s instructions as described previously^3^. Specifically, the supernatant from each plate was transferred to a fresh 96-well plate. The working solution was prepared according to the manufacturer’s instructions, and 60 μL was added to each well. The plates were then incubated for 30 min at room temperature on a shaker in dark. Absorbance was measured at 490 nm/600 nm using a microplate reader. LDH release was calculated using the following formula: LDH release rate (%) = (absorbance of treated sample -absorbance of sample control well) / (absorbance of maximum enzyme activity of cells -absorbance of sample control well) × 100.

***Measurement of ROS***

To measure ROS, cells of interest were incubated at 37°C for 30 min after treatment with 10 μM DCFH-DA from the ROS Assay Kit (Cat. #S0033S, Beyotime). Cells were then resuspended with PBS before ROS levels were detected using the Zeiss fluorescence microscope. The data of images were then statistically analyzed using ImageJ 1.53c (National Institutes of Health).

***Mitochondrial membrane potential (MMP) assays***

The analysis of MMP was carried out by using the Mitochondrial Membrane Potential Assay Kit with tetramethylrhodamine ethyl ester (TMRE) (Cat. #C2001S, Beyotime). All assays were described as previously^1^. The TMRE working solution was prepared according to the manufacturer’s instructions. The working solution of TMRE was added to the cells of interest and incubated at 37°C for 30 min and the cells were then centrifuged at 200 × g for 5 min, and the supernatant was discarded. The background of the TMRE working solution was removed by washing twice with a preheated medium. After discarding the supernatant, cells were resuspended in fresh PBS, and the fluorescence parameters were adjusted and photographed using a Zeiss fluorescence microscope. Analysis of the images was carried out using ImageJ 1.53c (National Institutes of Health).

***Propidium iodide (PI) staining***

Cells pre-treated for a specific time were stained with PI (10 μg/mL) in the dark for 30 min and incubated at 37°C. The cells were centrifuged at 200 × g for 5 min and resuspended in PBS. Cells were observed under the Zeiss fluorescence microscope, and images were captured. The data of images were then statistically analyzed using ImageJ 1.53c (National Institutes of Health, Bethesda, MD). In addition, PI positive cells were also measured by flow cytometry when necessary.

***Immunofluorescence assay***

Cells of interest were plated on confocal dishes or glass slides for 24 h, fixed with 4% paraformaldehyde (Cat. #P0099, Beyotime) for 15 min and incubated with Triton-100 (Cat. #P0096, Beyotime) immunostaining buffer for 10 min, followed by blocking with 5% bovine serum albumin (BSA) for 1 h at room temperature and incubation with primary antibodies overnight at 4°C. The cells were washed three times with the PBST buffer for 5 min before being incubated with Alexa Fluor fluorescent secondary antibody at room temperature for 1 h, and stained with DAPI at a concentration of 5 μg/mL for 10 min. Finally, images of the cells were captured using a Zeiss LSM 900 confocal microscope. To examine mitochondrial morphology, cells of interest were incubated with MitoTracker at a concentration of 200 nM/mL for 30 min at 37°C. Cell images were captured using a Zeiss LSM 900 META.

***Mitochondria isolation***

Mitochondria were isolated by using the Cell Mitochondria Isolation Kit (Cat. #C3601, Beyotime) as instructed by the manufacturer. The working solution was prepared according to the manufacturer’s instructions, and the cells were collected by centrifugation at 200 × g for 5 min. The residual medium was removed by washing with pre-cooled PBS, and the supernatant was discarded after centrifugation. The separation reagent provided by the kit was added, gently re-suspended, and incubated on ice for 10 min. The suspensions were further transferred to a homogenizer and homogenized. The homogenized liquid was then transferred into an EP tube and centrifuged at 1000 × g for 10 min at 4°C. The supernatants were carefully transferred into a fresh EP tube, centrifuged at 3500 × g for 10 min at 4°C, and the precipitates were collected as mitochondria. The mitochondrial lysates were mixed with 40 μL of 2 × loading buffer after 20 min incubation on ice. The samples were then denatured in a metal bath at 100°C for 5 min. The supernatants (the precipitates were mitochondrial part) were collected and centrifuged at 12000 × g for 10 min at 4°C to generate cytoplasmic proteins. Samples were prepared after protein determination using the BCA method.

***Seahorse cell mitochondrial stress test assay***

Mitochondrial respiration capacity of MM cell lines infecting IFI27 (Tet-on) lentivirus were detected by Seahorse Cell Mito Stress Test Assay. The Seahorse XFe96 Sensor Cartridge was hydrated overnight at 37°C in a non-CO₂ incubator using XF calibrant (Cat. #100840-000, Sigma). After the overexpression, cells were washed with XF Cell Mito Stress Test Assay Medium, which consisted of Seahorse XF RPMI Medium (Cat. #103756-100, Agilent) supplemented with D- (+) -Glucose solution (Cat. #G8644, Sigma) and L-glutamine (Cat. #G7513, Sigma). The medium was adjusted to pH 7.4 at 37°C and sterilized by filtration through a 0.2 μm filter (Cat. #FF251, Beyotime). Before the assay, the XFe96 Cell Culture Microplates (Cat. #103793-100, Agilent) was treated with 50 µg/mL Poly-D-lysine hydrobromide (Cat. #P7886, Sigma) for 20min, cells were the seeded at the XFe96 Cell Culture Microplates with the concentration of 5 × 10^4^ cells/90 μL per well for 1 h at 37 °C under CO₂-free conditions. Assay compounds were loaded into the hydrated sensor cartridge according to the manufacturer’s protocol: port A, 1 μM oligomycin (Cat. #HY-N6782, MedChemExpress); port B, 0.5 μM FCCP (Cat. #HY-100410, MCE); port C, 0.5 μM rotenone (Cat. #HY-B1756, MCE) and 1 μM antimycin A (Cat. #HY-107406, MCE). The assay was performed using the Agilent Seahorse XFe96 Analyzer (Agilent, USA), and data were analyzed with Seahorse Wave Desktop and Report Generator software (Agilent, USA).

***Depletion of mitochondria***

OPM2 and OCI-My5 cells at 50-60% confluence were infected with lentiviral Myc-Parkin for 72 h before being incubated with Puromycin to generate stable expressing cell lines. The stable cell lines were then seeded in 6-well plates in 4 mL media at a cell density of 10^6^ cells/mL. To induce extensive mitophagy, cells were incubated with 20 μM CCCP for three cycles and each cycle was 12 h. Each cycle is spaced at 12-h intervals. After that, the media were flushed, and the efficiency of mitochondrial depletion was determined by detecting mitochondrial proteins and staining with a MitoTracker. Cell viability was detected using the CCK8 method，and cells lacking mitochondria were used for subsequent experiments. Such as the infection of IFI27 lentivirus or N-GSDME and the treatment of DOX.

***CCK8 assay***

The cells were uniformly seeded in 96-well culture plate, and no less than 3 multiple Wells were set up in each group to ensure the repeatability of the experiment. The cells were treated according to the experimental design, and then the culture plate was incubated in 37℃, 5% CO₂ constant temperature incubator until the detection time. At the end of the culture, 10 μL CCK-8 detection reagent was added to each well, and the culture plate was gently shaken to fully mix with the culture medium to avoid bubble formation. Subsequently, the 96-well plates were incubated in a 5% CO₂ incubator at 37°C in the dark for 1-2 h. After incubation, the culture plates were removed, and the absorbance of each well was measured at a wavelength of 450 nm using a microplate reader. The cell viability was evaluated according to the OD value of each treatment group and blank control group, and the data of each group were statistically analyzed by Graphpad Prsim software.

***Transmission electron microscopy (TEM)***

Cells were processed before TEM analysis as described previously^3^. An appropriate amount of IFI27 lentivirus was added dropwise to HeLa cells in a 6-well plate at 50-60% confluency and incubated overnight at 37°C with 5% CO_2_. After replaced with fresh medium (DMEM, 10% FBS), and the cells were further cultured for another 60 h. After removal of the medium cells were incubated with electron microscopy fixative (Cat. #BL911B, Biosharp) in dark at room temperature on a shaker for 5 min. The fixative was discarded after short spins, cells were gently resuspended in fresh electron microscopy fixative then gently scraped in one direction using a cell scraper and transferred to a 15-mL centrifuge tube using a Pasteur pipette and further fixed at dark at room temperature for 30 min. Finally, subcellular structure was analyzed on a Hitachi HT7700 TEM (Tokyo, Japan).

**Supplemental Figures and legends:**

**Supplemental Figure S1. IFI27 impairs mitochondria and disrupts its integrity in MM cells.** OCI-My5 cells were infected with lentivirus IFI27 for 72 h, cells were then prepared for RNA-seq, followed by bubble diagram of GO analysis.

**Supplemental Figure S2. IFI27 impairs mitochondria and disrupts its integrity in MM cells.** A, MM cell lines OCI-My5 and OPM2 were infected with lentiviral IFI27 for 72 h, followed by TMRE staining, the TMRE MFI was analyzed (n =5). B-C, OCI-My5 cell was infected with IFI27 lentivirus followed by DCFH-DA staining (B) and statistical analysis (C) (n = 5). Scale bars, 20 μm. D-E, OPM2 cell stably infected with an IFI27 (Tet-on) lentiviral plasmid was treated with N-acetyl cysteine (NAC, 5 mM) for 5 h. Cells were further treated with doxycycline (DXC, 1 μg/mL) for 48 h, followed by statistical analysis of DCFH-DA and PI staining (D) and statistical analysis of fluorescence ratio (E) (n =5). Scale bars, 50 μm. F, OCI-My5 and OPM2 stably infected with an IFI27 (Tet-on) lentiviral plasmid were treated with N-acetyl cysteine (NAC) for 5 h. Cells were further treated with doxycycline (DXC) for 48 h, followed by IB assays. ***, *P* < 0.001. EV, empty virus.

**Supplemental Figure S3. Mitochondria are required for IFI27-induced MM cell pyroptosis.** A, OPM2 cells were infected with lentiviral Myc-Parkin. Mitophagy was triggered in OPM2 cells by expressing Parkin and treatment with CCCP (20 μM). Cells were stained with MitoTracker to test the effect of mitochondrial deficiency. Exposure time of all cell images: 200 ms. Scale bars, 100 μm. B, OPM2 and OCI-My5 cells stably infected with lentiviral Myc-Parkin were treated with 20 μM CCCP at 90%-100% confluency in complete medium for 3 cycles and each cycle was 12 h. After mitochondrial depletion, cells were then cultured in CCCP-free media and further infected lentiviral IFI27 for 48 h, followed by staining with TMRE before being analysis on a fluorescence microscope. C, The statistical analysis on the TMRE staining cells. Scale bar, 50 μm. ***, *P* < 0.001. n.s, not significant. EV, empty virus. OE, overexpression.

**Supplemental Figure S4. Mitochondria are required for IFI27-induced MM cell pyroptosis.** A, MM cells viability was determined by the CCK8 colorimetric assay after cells were treated with CCCP (20 μM) for 24 hr, or infected with Parkin lentivirus. Statistical analysis using GraphPad Prism software (n = 5). B-C, OPM2 and OCI-My5 cells stably infected with lentiviral Myc-Parkin were treated with 20 μM CCCP at 90%-100% confluency in complete medium for 3 cycles and each cycle was 12 h. After 3 cycles of treatment with CCCP, cells were further infected lentiviral IFI27 for 48 h, followed by cell vibility assay (B) or staining with PI before being analysis on a flow cytometer (C). Mitodepletion: Parkin + CCCP treatment. **, *P* < 0.01; ***, *P* < 0.001; n.s, not significant. EV, empty virus. OE, overexpression.

**Supplemental Figure S5. Mitochondria are required for IFI27- and DOX- induced MM cell pyroptosis. A**, OPM2 and OCI-My5 cells stably infected with lentiviral Myc-Parkin were treated with 20 μM CCCP at 90%-100% confluency in complete medium for 3 cycles and each cycle was 12 h. After mitochondrial depletion, cells were then cultured in CCCP-free media and further infected lentiviral IFI27 for 48 h. Pyroptosis morphology was determined by a phase-contrast microscope followed by analyze the rate of pyroptosis (n = 6). B, After being depleted mitochondria, cells were treated with DOX for 24 h, followed by pyroptotic analysis on a phase-contrast microscope followed by analyze the rate of pyroptosis. **, *P* < 0.01; ***, *P* < 0.001; n.s, not significant. EV, empty virus. OE, overexpression.

**Supplemental Figure S6. IFI27 is required for cell pyroptosis induced by DOX.** OCI-My5 and OPM2 cells were infected with lentiviral sgRNA of IFI27 as indicated, followed by DOX treatment for 24 h, the IB assay were performed (A) and the proportion of pyroptotic cell were analyzed (B) (n =5). **, *P* < 0.01; ***, *P* < 0.001; n.s, not significant. EV, empty virus.

***References***

1.	Wang Y, Cui Y, Liu Z, Liu L, Huang Z, Wang Q*, et al.* Acevaltrate overcomes myeloma resistance to bortezomib via pyroptosis by promoting BAX translocalization to mitochondria. *Eur J Pharmacol* 2025, **996:** 177572.

2.	Sun Y, Jiang L, Zhang Z, Zhu R, Liang J, Liu Z*, et al.* RNF6 Inhibits Lung Adenocarcinoma Cell Proliferation by Promoting Cyclin D2 Degradation. *Mol Cancer Res* 2025, **23**(5)**:** 426-437.
